# Investigating mitochondrial fission, fusion, and autophagy in retinal pigment epithelium from donors with age-related macular degeneration

**DOI:** 10.1038/s41598-022-26012-5

**Published:** 2022-12-16

**Authors:** Cody R. Fisher, Adam A. Shaaeli, Mara C. Ebeling, Sandra R. Montezuma, Deborah A. Ferrington

**Affiliations:** 1 Department of Ophthalmology and Visual Neurosciences, University of Minnesota, Minneapolis, MN 55455 USA; 2 Graduate Program in Biochemistry, Molecular Biology, and Biophysics, University of Minnesota, Minneapolis, MN 55455 USA; 3 Stem Cell Institute, University of Minnesota, Minneapolis, MN 55455 USA; 4 Undergraduate program in Biochemistry, Molecular Biology, and Biophysics, University of Minnesota, Minneapolis, MN 55455 USA; 5 Doheny Eye Institute, Pasadena, CA 91103 USA

**Keywords:** Mechanisms of disease, Proteins, Mitophagy

## Abstract

Age-related macular degeneration (AMD) is the leading cause of irreversible blindness in developed countries, characterized by the death of retinal pigment epithelial (RPE) cells and photoreceptors. Previous studies report an accumulation of damaged and dysfunctional mitochondria in RPE of human donors with AMD. Understanding how damaged mitochondria accumulate in AMD is an important step in discovering disease mechanisms and identifying therapeutic targets. In this report, we assessed mitochondrial fission and fusion by quantifying proteins and measured mitochondrial autophagy (mitophagy) via protein analysis and advanced imaging techniques using mitochondrial targeted mKeima in primary human RPE from donors with or without AMD. We report disease-specific differences in mitochondrial proteins that regulate fission, fusion, and mitophagy that were present at baseline and with treatments to stimulate these pathways. Data suggest AMD RPE utilize receptor-mediated mitophagy as a compensatory mechanism for deficits in the ubiquitin-mediated mitophagy pathway. These changes in mitochondrial homeostasis could lead to the buildup of damaged and dysfunctional mitochondria observed in the RPE of AMD donors.

## Introduction

Age-related macular degeneration (AMD) is the leading cause of irreversible blindness in developed countries. This multifactorial disease involves complex genetic and environmental factors whose effects accelerate with age^1,2^. AMD affects 30% of individuals 75–85 years of age, with a global estimate of 128 million cases in 2020 and 288 million cases by 2040, a consequence of the world’s increasing elderly population^3,4^. The two forms of AMD include “wet” and “dry”, with wet AMD caused by the abnormal growth of blood vessels into the retina. While wet AMD is less common, it has a number of effective therapies to halt or prevent vision loss^5^. Dry AMD is the most common form of the disease, accounting for approximately 85% of all AMD cases^6^. Vision loss associated with dry AMD is caused by the death of retinal pigment epithelium (RPE) and photoreceptors. RPE are essential for maintaining a healthy retina as they are responsible for key functions, such as the transport of nutrients to photoreceptors and the directed secretion of growth factors^7^. Currently, there are no effective treatments for dry AMD, due to our incomplete understanding of the cellular events causing disease pathology. Thus, there remains an urgent need to identify the underlying mechanisms causing AMD in order to successfully develop therapeutic interventions.

One of the prevailing hypotheses is that RPE mitochondrial defects drive AMD pathology^8^. This hypothesis is supported by numerous studies in human retina from eye bank donors. Analysis of electron microscopy images found donors with AMD had significantly fewer mitochondrial number, reduced surface area, and an altered cristae morphology^9^. Additionally, proteomic studies of human RPE tissue found an altered mitochondrial proteome, with multiple proteins in the electron transport chain decreasing in AMD RPE^10,11^. There are also reports of increased mitochondrial DNA damage with progression of AMD severity^12,13^. Consistent with the mitochondrial defects observed in tissue, cultures of human primary RPE showed significantly decreased mitochondrial function in RPE from donors with AMD^14–16^. Use of primary RPE cultures from human donors has also shown dysfunctional autophagy and increased mitochondrial damage^15^. Taken together, these findings suggest that mitochondrial dysfunction is a contributing factor to AMD pathology.

We tested the hypothesis that dysfunctional mitochondria accumulate in AMD due to defects in the processes that maintain a healthy mitochondrial population. We investigated three major pathways of mitochondrial homeostasis (mitochondrial fission, fusion, and mitophagy) to investigate mechanisms underlying mitochondrial dysfunction in AMD. Primary RPE cultures from donors with dry AMD (AMD) or without disease (No AMD) were treated with 2-[2-[4-(trifluoromethoxy)phenyl]hydrazinylidene]-propanedinitrile (FCCP) or cobalt chloride (CoCl_2_). FCCP is a mitochondrial uncoupler, leading to rapid loss of membrane potential, stimulating fission and mitophagy^17^. Treatment with CoCl_2_ was used to simulate hypoxia and induce receptor-mediated mitophagy^18–22^. Proteins that regulate pathways of mitochondrial homeostasis were quantified using immunoblotting. These measurements were coupled with live-cell imaging using mKeima-mito, a mitochondrial targeted fluorescent protein that allows for real-time monitoring of mitophagy.

In this study, we report disease-specific differences in mitochondrial proteins that regulate fission, fusion, and mitophagy that were present at baseline and with treatments that stimulate these pathways. These data suggest that changes in mitochondrial homeostasis could lead to the buildup of damaged and dysfunctional mitochondria observed in RPE of AMD donors.

## Methods

### Cell culture

RPE cells from de-identified donor eyes were obtained from Lions Gift of Sight (formerly known as Minnesota Lions Eye Bank) in Saint Paul, MN. Donor demographics are available in Supplementary Table 1. Donor eyes were obtained with the informed consent of the donor or donor’s family for use in medical research in accordance with the Declaration of Helsinki. Tissue handling, storage, and donor exclusion criteria are as outlined^23,24^. Our study utilizes donors without disease as age-matched controls and donors with dry AMD. The Minnesota Grading System (MGS) was used to classify donor eyes into No AMD (MGS1) and AMD (MGS2 and MGS3)^23,24^. Evaluation for MGS stages was determined by a Board Certified Ophthalmologist (Dr. Sandra R. Montezuma). Primary RPE were cultured as outlined^17^. Cells in passage 3 were used for all experiments. All experimental protocols were approved by the Office of Biotechnology Activities Oversight Institutional Biosafety Committee (UMN, IBC Code Number: 1706-34907H) and carried out in accordance with relevant guidelines and regulations.

RPE were treated with either 5 μM FCCP or 250 μM CoCl_2_. Optimal dose of FCCP was determined in a previously published methods article^16^. Optimal dose of CoCl_2_ was chosen based on preliminary experiments (Supp. Figure 9).

#### AAV5-mKeima-mito

Adeno-associated virus serotype 5 (AAV5) mKeima-mito was produced by the University of Minnesota Viral Vector and Cloning Core. RPE were infected, imaged, and analysis done following a previously published method^17^. In summary, RPE were plated at subconfluent conditions before infection with AAV5-mKeima-mito. Infection and expression efficiency reached 80% ± 10%.

### Western immunoblotting

RPE were plated at confluence on Synthemax (Corning) coated 12-well plates and grown for at least 2 days before being treated with either 5 μM FCCP or 250 μM CoCl_2_. Cells were lysed with RIPA Buffer (Thermo Fisher) and protein concentration determined using a Bicinchoninic acid protein assay (Thermo Fisher). Protein (10 μg) was resolved on stain-free SDS-PAGE gels as described previously^25^. Representative immunoblots are shown (Supplementary Figs. 1–4). A table of antibodies used in this report is available in Supplementary Table 2. Data was calculated relative to protein load by using the stain free PVDF image, as well as relative to a standard protein sample included on each gel.

### Statistical analysis

Fold change data was log transformed (log2) before statistical analysis. All statistical tests used an alpha of 0.05. A Grubb’s test was used to remove outliers. Normality was tested using the Shapiro–Wilk test. If data was normally distributed, an unpaired t-test was used to determine significance between AMD and No AMD. For data with a non-normal distribution, a Mann–Whitney test was used to determine significance between AMD and No AMD. To determine significance from untreated controls, a one-sample t-test or Wilcoxin signed rank test was used for normally or non-normally distributed data, respectively. mKeima-mito data (Figs. 5 and 6) was assessed using a 2-way ANOVA with treatment and disease effects and a Sidak’s multiple comparison to determine significance between No AMD and AMD RPE. Statistical analysis was performed using GraphPad Prism 9. Data presented is a mean ± SEM.

## Results

### Experimental design

**Figure 1:**
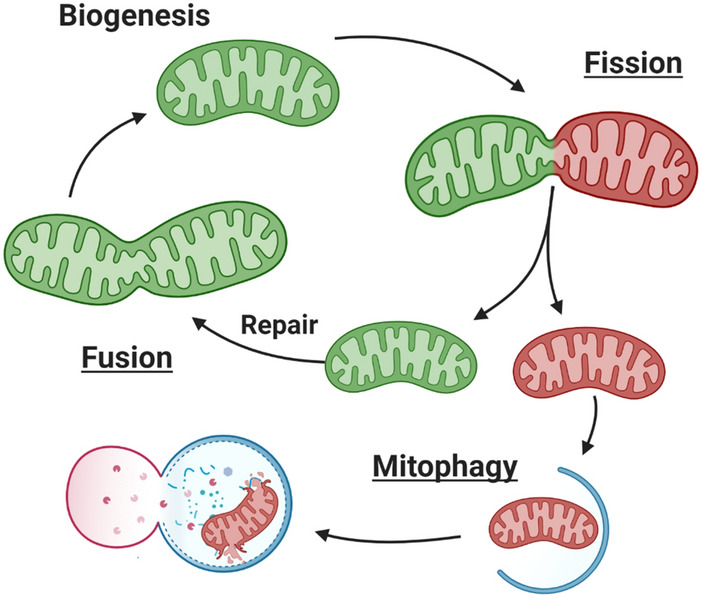
Overview of Mitochondrial Homeostasis. Cells maintain a healthy network of mitochondria through Biogenesis, Fission, Fusion, and Mitophagy. Under stress or damage, often resulting in mitochondrial membrane depolarization, dysfunctional mitochondria (red) are separated (Fission) from the healthy pool (green) and targeted for degradation. To maintain the healthy pool of mitochondria, networks fuse to mix membranes, proteins, and mtDNA (Fusion). Damaged fragments of mitochondria are separated from healthy networks and degraded in a specialized type of autophagy involving mitochondrial engulfment by LC3 associated-autophagosomes (Mitophagy). After engulfment, the autophagosome (blue) fuses with a lysosome (pink) and lysosomal enzymes degrade the mitochondria. Mitochondria are repaired and replenished by generating new mitochondrial membranes, proteins, and mtDNA through a coordinated effort of nuclear and mitochondrial DNA gene expression (Biogenesis). Figure produced in BioRender.

Healthy mitochondria are maintained through a combination of fission, fusion, mitochondrial autophagy (mitophagy), and biogenesis (Fig. 1). Fission, stimulated by changing cellular conditions such as mitochondrial depolarization and oxidative stress, is required to cull damaged mitochondria from the healthy pool. Undamaged or repaired mitochondria fuse with other healthy mitochondria, increasing mitochondrial efficiency^26^. Damaged mitochondria are removed via mitophagy, a process that involves sequestering of target mitochondria within autophagosomes which then fuse with lysosomes where degradation occurs^22^. Biogenesis, while not investigated in our study, completes the circuit by supplying lipids, proteins, and DNA to replenish the mitochondrial population.

**Figure 2:**
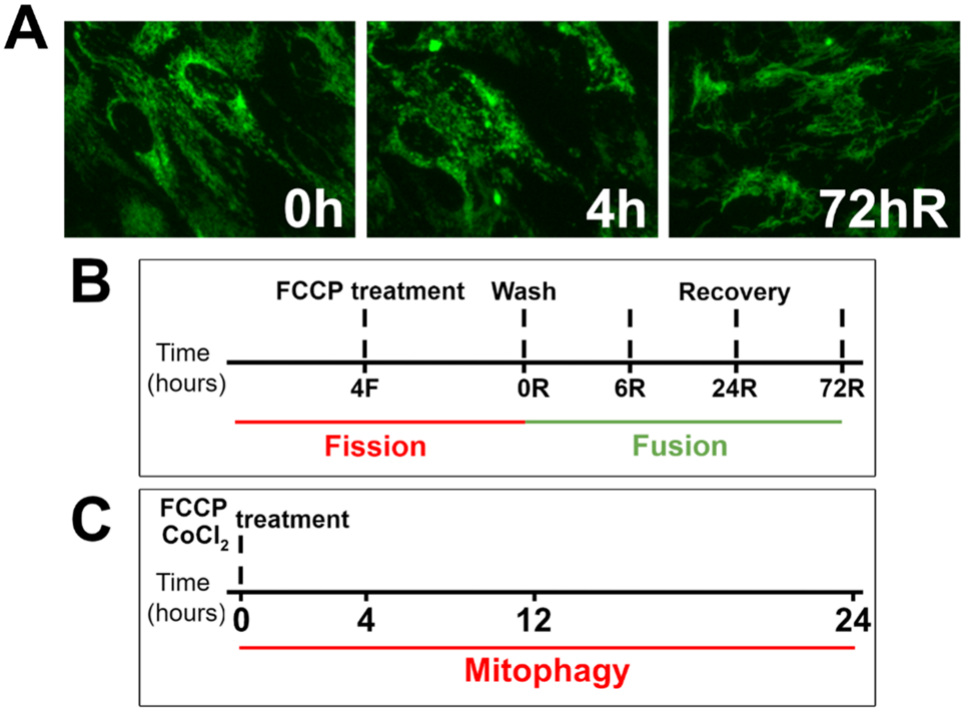
Experimental design for measuring fission, fusion, and mitophagy. (**A**) Representative images of neutral mKeima-mito showing untreated cells (0 h), mitochondrial fragmentation (fission, 4 h 5μM FCCP) and recovery (fusion, 72 h recovery, 72R). (**B**, **C**) Experimental design with time points for fission and fusion (**B**) and mitophagy (**C**) experiments.

We previously characterized the use of FCCP in human primary RPE to stimulate fission and mitophagy^17^. Representative images of mitochondrial fragmentation and recovery after removal of FCCP are shown in Fig. 2A. Mitochondrial fission and fusion were quantified after FCCP treatment and through the recovery phase after removal of FCCP by washing cells with fresh culture media (Fig. 2B). In a separate experiment, short term and long term mitophagy responses were quantified in cells treated with FCCP or CoCl_2_ for up to 24 h (Fig. 2C). Proteins regulating mitochondrial homeostasis were quantified and time-lapse live-cell imaging using mKeima-mito was performed using this experimental protocol. Two main questions are addressed by our experimental design; (i) is there a treatment response relative to untreated controls and (ii) is there a difference at baseline or in response to treatment between RPE from No AMD and AMD donors?

### Fission and fusion

**Figure 3:**
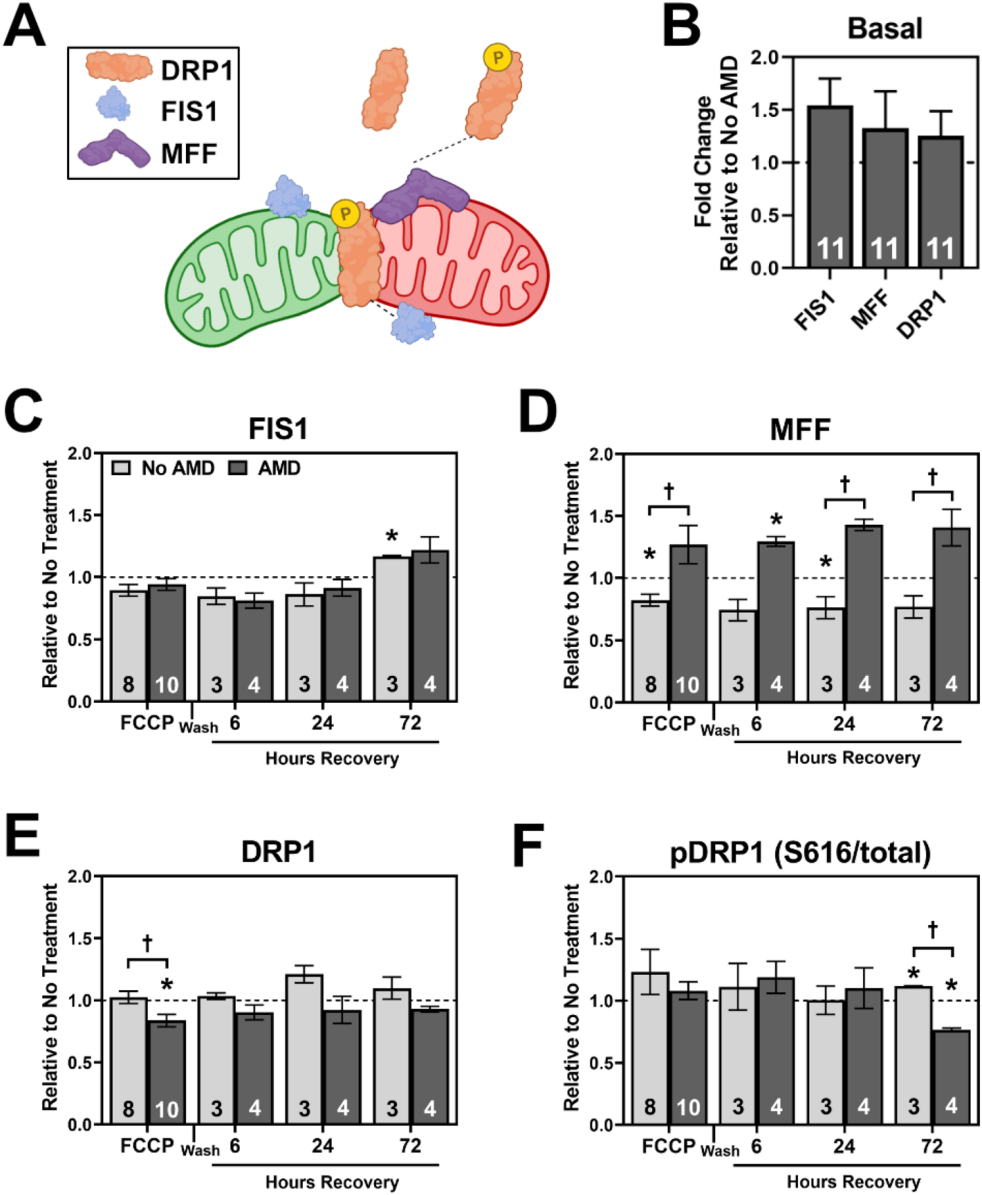
Response of Mitochondrial Fission Machinery to FCCP stress and recovery. (**A**) Summary of proteins involved in mitochondrial fission. Mitochondrial fission factor (MFF) and mitochondrial fission 1 protein (FIS1) activate under conditions of mitochondrial stress, recruiting dynamin-1-like protein (DRP1) to the site of fission. Once active by phosphorylation (P), pDRP1 divides the healthy (green) from the damaged (red) mitochondria. (**B**) Basal content of fission proteins in AMD RPE relative to No AMD RPE. (**C**–**F**) Quantification of fission proteins FIS1 (**C**), MFF (**D**), DRP1 (**E**), and pS616-DRP (**F**) after 4 h FCCP and recovery time points. All data shown is mean ± SEM. Number of samples for each experiment are provided in the bars. * denotes significance (*p* < 0.05) from no treatment controls (dashed line). † denotes significance (*p* < 0.05) between No AMD and AMD RPE. Panel A produced in BioRender.

Under conditions of mitochondrial damage and depolarization, fission proteins MFF and FIS1 recruit DRP1 to the site of fission^26^ (Fig. 3A). Once activated by phosphorylation, DRP1 begins to bisect the mitochondria into two pieces thereby isolating damaged mitochondria away from the healthy pool. FCCP treatment was used to stimulate fission, with lysates collected as described (Fig. 2B). Fission proteins FIS1, MFF, DRP1, and activated DRP1 (pS616-DRP1) were quantified via Western immunoblotting. Under basal conditions there was no AMD-dependent difference in content of fission proteins (Fig. 3B). After FCCP, abundance levels of FIS1 significantly increased in No AMD RPE after 72 h of recovery (*p* = 0.026) (Fig. 3C). For MFF, a dramatic disease-related effect was observed (Fig. 3D). While there was an overall decrease in MFF for No AMD RPE, MFF content was increased at each time point in AMD RPE. With FCCP stress, total abundance of DRP1 was significantly lower for AMD RPE (*p* = 0.012), which was significantly different than the response in No AMD (*p* = 0.020) (Fig. 3E). The content of pDRP1-S616 (an indicator of activation) relative to total DRP1 was significantly increased in No AMD and significantly decreased in AMD after 72 h recovery (Fig. 3F, *p* = 0.001).

**Figure 4:**
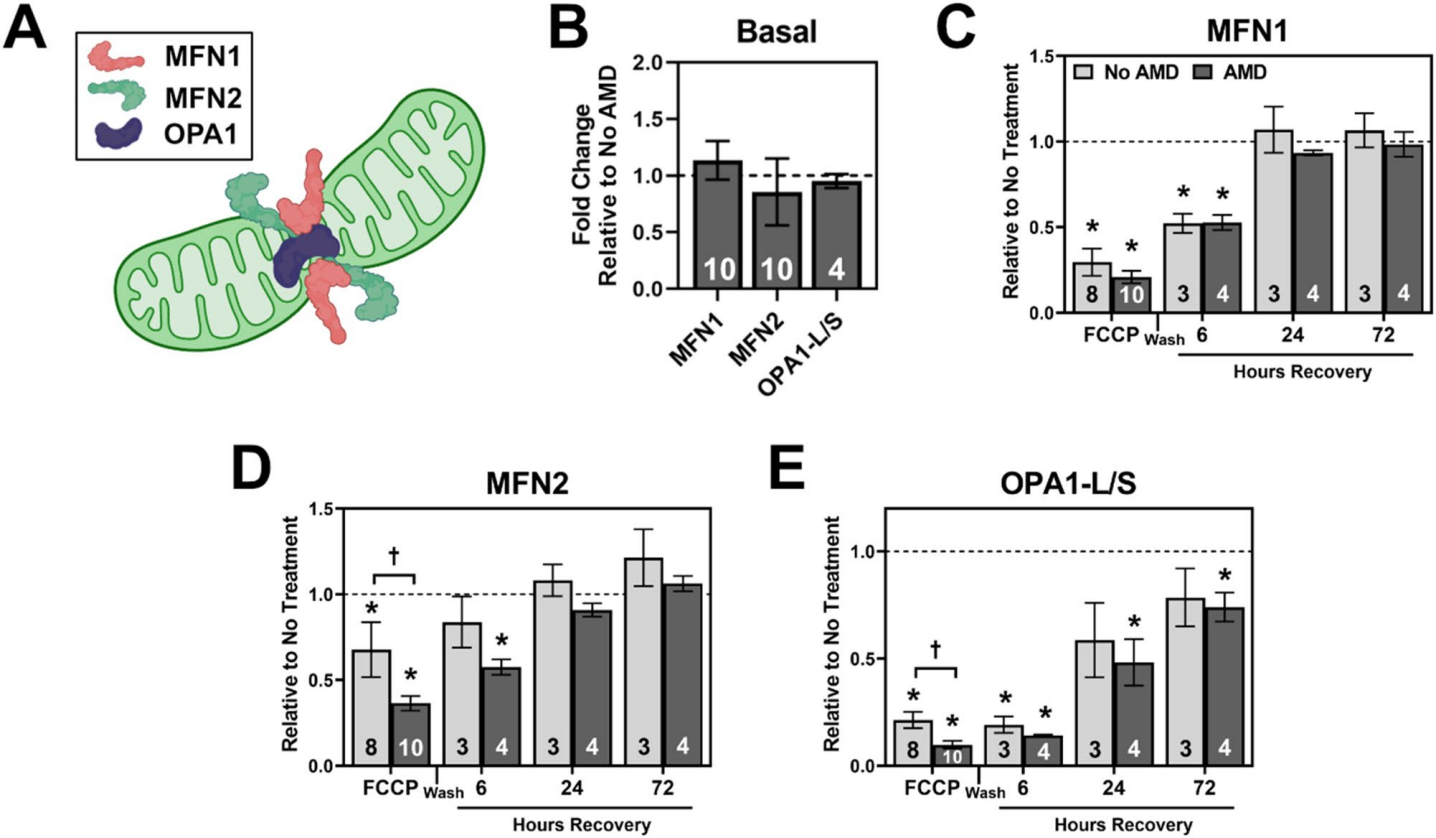
Response of Mitochondrial Fusion Machinery to FCCP stress and recovery. (**A**) Schematic of mitochondrial fusion. Healthy mitochondria (green) fuse the outer mitochondrial membranes via mitofusin-1 (MFN1), mitofusin-2 (MFN2), and the inner mitochondrial membrane via dynamin-like 120 kDa protein (OPA1). (**B**) Basal content of fusion proteins in AMD RPE relative to No AMD RPE. (**C**–**E**) Quantification of fusion proteins MFN1 (**C**), MFN2 (**D**), OPA1-L/S (**E**) after 4 h FCCP and recovery time points. All data shown is mean ± SEM. Number of samples for each experiment is provided in the bars. * denotes significance (*p* < 0.05) from no treatment controls (dashed line). † denotes significance (*p* < 0.05) between No AMD and AMD RPE. Panel A produced in BioRender.

In parallel with quantification of fission proteins, the fusion proteins MFN1, MFN2, and OPA1 were quantified. These three proteins lead to the fusion of both outer and inner mitochondrial membranes^26^(Fig. 4A). There were no significant differences in protein content for No AMD and AMD RPE under basal conditions (Fig. 4B). For MFN1, the magnitude and direction of change in content was similar for both No AMD and AMD RPE (Fig. 4C). MFN1 content was significantly lower with FCCP (No AMD, *p* = 0.001; AMD, *p* < 0.001) and at 6 h of recovery (No AMD, *p* = 0.028; AMD, *p* = 0.004) but returned to baseline by 24 h of recovery. Similar to MFN1, the content of MFN2 decreased with FCCP but returned to basal levels after 24 h of recovery (Fig. 4D). Disease-related differences include the lower MFN2 content in AMD donors after 4 h of FCCP (*p *= 0.043). OPA1 had both a long form (OPA1-L) and a short form (OPA1-S) visible using immunoblotting. OPA1-L promotes mitochondrial fusion while OPA1-S promotes mitochondrial fission^27,28^. Both bands of OPA1 were quantified separately (Supplementary Fig. 5) and reported as the ratio of OPA-L/S (Fig. 4E). Content of OPA1-L/S significantly decreased following 4 h FCCP treatment in No AMD (*p* = 0.014) and AMD RPE (*p* = 0.001) (Fig. 4E). However, RPE from No AMD donors had significantly more OPA1-L/S than AMD donors after 4 h of FCCP treatment (*p* = 0.039). While OPA1-L/S began to increase during the recovery time points for both groups of RPE, AMD remained significantly decreased at 24 (*p* = 0.027) and 72 h of recovery (*p* = 0.038). Taken together, these data highlight subtle disease-specific changes in fission and fusion machinery.

### Quantification of mitophagy

**Figure 5:**
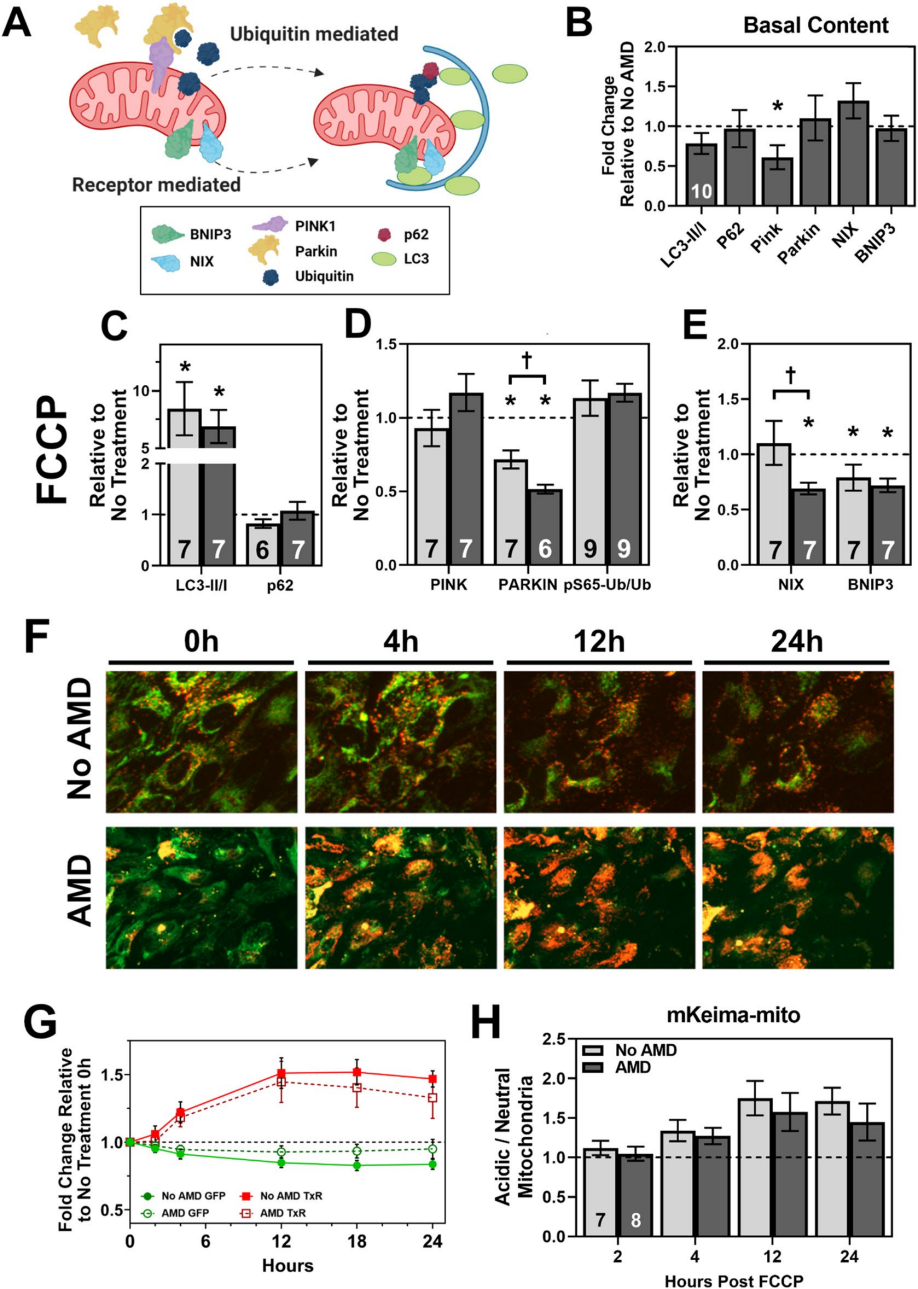
Assessment of mitophagy after FCCP induced stress. (**A**) Summary of ubiquitin-mediated and receptor-mediated mitophagy pathways. Membrane depolarization leads to PTEN-induced kinase 1 (PINK1) activation, leading to PARKIN (E3 ubitiquin-ligase) ubiquitination of outer membrane proteins which initiates autophagosome formation. Receptor-mediated mitophagy proteins BCL2/adenovirus E1B 19 kDa protein-interacting protein 3 (BNIP3) and BCL2/adenovirus E1B 19 kDa protein-interacting protein 3-like (NIX) are stimulated by PINK1, as well as other stressors including hypoxia. BNIP3 and NIX directly interact with autophagosome machinery to induce mitophagy. (**B**) Basal content of mitophagy proteins in AMD RPE relative to No AMD (dashed line). (**C**, **D**) Quantification of LC3-II/I and p62 (**C**), ubiquitin-mediated mitophagy proteins PINK, PARKIN, and pS65-Ub / total Ub (**D**), or receptor-mediated mitophagy proteins NIX and BNIP3 (**E**) proteins after 4 h of FCCP treatment in No AMD (light bar) or AMD (dark bar). (**F**) Representative images of mKeima-mito from No AMD (top) and AMD (bottom) RPE. (**G**) Traces of neutral (green, circles) and acidic (red, squares) after treatment with FCCP for No AMD (filled symbols, solid line) and AMD (open symbols, dashed line) RPE. (**H**) Ratio of acidic to neutral mitochondria in primary RPE from No AMD or AMD donors. All data shown is mean ± SEM. Number of samples for each experiment is provided in the bars. Results of 2-way ANOVA are treatment effect (p < 0.01) and disease effect (*p* = 0.40). Panel A produced in BioRender.

Two main pathways of mitophagy, the ubiquitin-mediated (PINK1/Parkin) and receptor-mediated mitophagy (BNIP3, NIX) pathways were quantified (Fig. 5A). Under healthy conditions, the kinase PINK1 is degraded within the mitochondria^22,29^. However, under conditions of stress and depolarization, PINK1 remains in the outer mitochondrial membrane and activates PARKIN, an E3-ubiquitin ligase. PARKIN then adds a specific phosphorylated ubiquitin (pS65-Ubiquitin) to outer mitochondrial membrane proteins, leading to degradation by the proteasome and initiation of mitophagy. Receptor-mediated mitophagy proteins NIX and BNIP3 can be activated by PINK1, as well as conditions of hypoxia and other stressors^22^. Upon activation, NIX and BNIP3 directly interact with autophagosomes through p62 and LC3, without ubiquitin, to initiate mitophagy.

We quantified cellular autophagy markers (LC3 and p62), as well as proteins from ubiquitin-mediated mitophagy (PINK, Parkin, and pS65-Ubiquitin (pS65-Ub)) and receptor-mediated mitophagy (Nix and BNIP3) (Fig. 5A). Under basal conditions, AMD RPE had ~ 50% less PINK content (*p* = 0.015) (Fig. 5B). Treatment with FCCP had no effect on p62 but led to an approximate eightfold increase in LC3-II / LC3-I content, an indication that cellular autophagy is robustly increased in both No AMD and AMD RPE (Fig. 5C). While there were no significant differences in PINK1 or pS65-Ubiquitin content after FCCP, Parkin content was significantly decreased in both groups (No AMD, *p* = 0.007; AMD, *p* < 0.001) but was significantly lower in AMD RPE (*p* = 0.015) (Fig. 5D). The receptor-mediated proteins were also quantified, with AMD RPE having significantly decreased NIX content compared with No AMD RPE (*p* = 0.040). Both No AMD (*p* = 0.002) and AMD (*p* = 0.029) had significantly decreased BNIP3 content (Fig. 5E). To assess the effects of sustained FCCP on mitophagy proteins, we also quantified proteins after 24 h of FCCP (Supplementary Fig. 6). Nearly all proteins returned to basal levels, indicating both cellular autophagy and mitophagy had slowed relative to the four hour treatment data. This response agrees with previously published findings that membrane potential returns to baseline at 24 h FCCP treatment^17^.

To determine if the disease-dependent differences in mitophagy machinery have functional effects, we quantified cellular mitophagy using mKeima-mito. A recent publication from our laboratory provides a detailed summary of the quantification of mitophagy using mKeima-mito^17^. Briefly, mKeima is a fluorescent protein that shifts excitation wavelengths based on pH of the local environment. Mitochondria in the cytoplasm are in a neutral pH environment, but upon fusion with the lysosome are contained within an acidic environment. This change in local pH during mitophagy allows for the imaging of mKeima using two fluorescent channels. Fusing mKeima to a mitochondrial targeting sequence allows for mitophagy to be quantified as a ratio of neutral (green) cytoplasmic mitochondria to acidic (red) lysosomal mitochondria. Representative images of primary RPE expressing mKeima-mito treated with FCCP are shown (Fig. 5F). Individual neutral (green) and acidic (red) channels for both No AMD and AMD RPE treated with FCCP are shown (Supplementary Fig. 7).

Treatment with FCCP leads to fragmentation of mitochondria and induction of mitophagy, as seen as an increase in acidic (red) and slight decrease in neutral (green) traces (Fig. 5G). After calculating the ratio of acidic to neutral mitochondria, an increase in mitophagy is visualized as an increase in the ratio (Fig. 5H). This ratio shows FCCP treatment induced a significant increase in mitophagy for both No AMD and AMD RPE (p = 0.001). Of note, AMD RPE were consistently lower at each time point than No AMD RPE (~ 6–18%), suggesting a slight decrease in mitophagy, though this difference was not statistically significant.

In addition to FCCP treatment, RPE were treated with FCCP and had chloroquine (CQ) added 2 h before lysis to prevent autophagosome and lysosomal fusion^30^. Treatment with CQ prevents autophagosome-lysosomal fusion, effectively stalling cellular autophagy (Mauthe et al.^31^). This allows for quantification of autophagy flux, an important control to include when cellular autophagy is very rapid. Overall, the results from FCCP treatment with and without CQ provided similar answers except for the results for BNIP3 (Supplementary Fig. 8). BNIP3 content was increased in No AMD RPE relative to FCCP alone, and was significantly different than AMD RPE (*p* = 0.016).

### Cobalt chloride and mitochondrial homeostasis

As AMD RPE had significant differences in receptor-mediated mitophagy proteins in response to FCCP, we wanted to further investigate these pathways. Additionally, FCCP causes rapid depolarization of the mitochondrial membrane and therefore is not physiologically relevant to AMD disease mechanisms. Receptor-mediated mitophagy proteins, BNIP3 and Nix, are activated by hypoxia^32^. To stimulate receptor-mediated mitophagy with a more physiologically relevant stress, we simulated hypoxia via HIF1α stabilization with cobalt chloride (CoCl_2_). To determine optimal dose, we measured cell viability and mitochondrial membrane potential (Supplementary Fig. 9). As the goal of this treatment was to induce a mitochondrial stress response without cell death, we chose a dose of 250 μM CoCl_2_.

**Figure 6:**
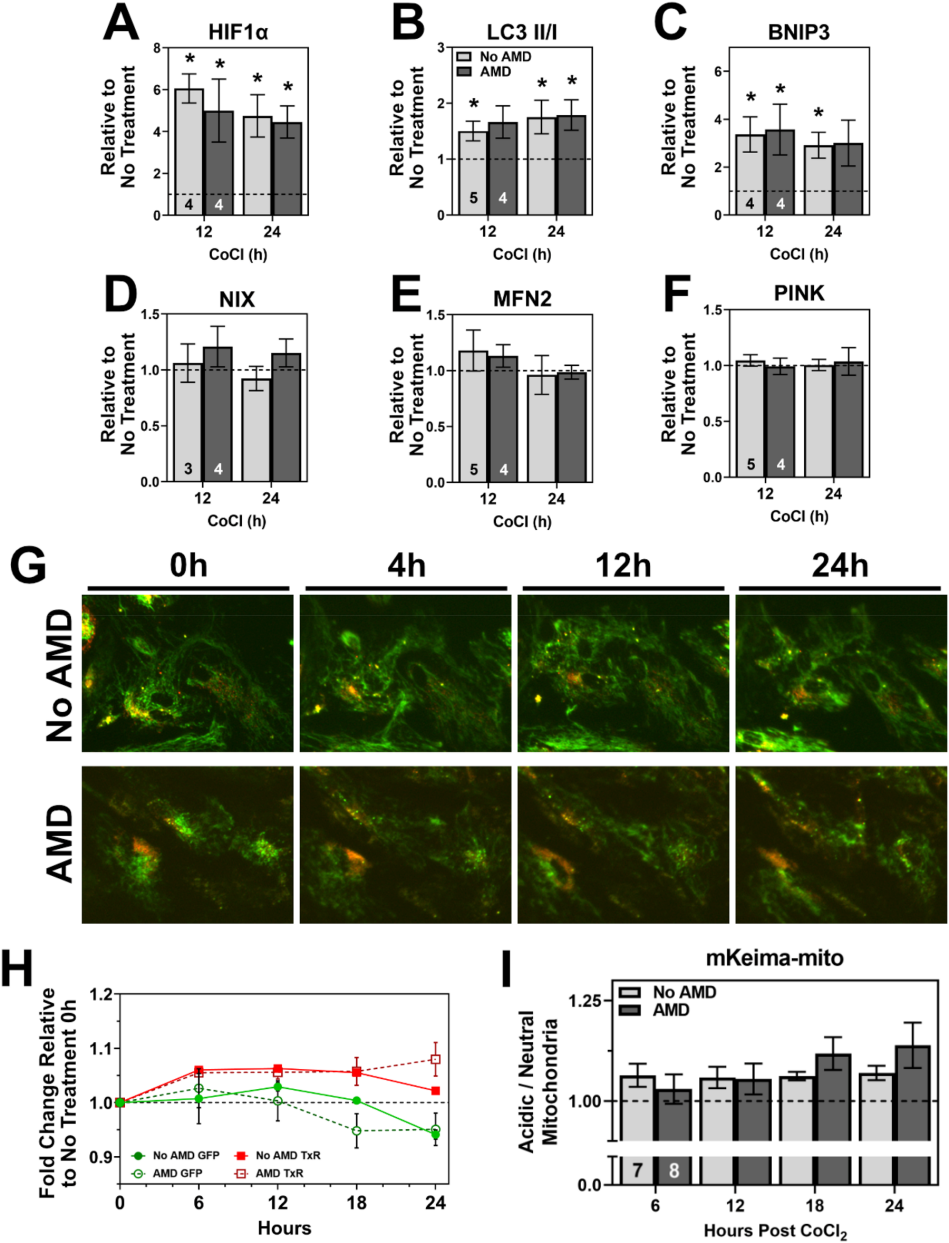
RPE response to Cobalt Chloride induced hypoxia. (**A**–**F**) Quantification of proteins after 12 or 24 h of 250 µM CoCl_2_ treatment for HIF1α (**A**), LC3-II/I (**B**), BNIP3 (**C**), NIX (**D**), MFN2 (**E**), and PINK (**F**). (**G**) Representative mKeima-mito images from No AMD (top) and AMD (bottom) RPE after treatment with CoCl_2_. (**H**) Traces of neutral (green, circles) and acidic (red, squares) after treatment with CoCl_2_ for No AMD (filled symbols, solid line) and AMD (open symbols, dashed line) RPE. (**I**) Ratio of acidic to neutral mitochondria in primary RPE from No AMD or AMD donors. All data shown is mean ± SEM. Number of samples for each experiment is provided in the bars. Results of 2-way ANOVA are treatment effect (*p* = 0.17) and disease effect (*p* = 0.63).

CoCl_2_ treatment led to an expected increase in HIF1α content at 12 and 24 h in RPE irrespective of disease status (Fig. 6A). Treatment also led to a significant upregulation of autophagy, as indicated by the increased content of LC3-II/I in both No AMD and AMD RPE at 12 (No AMD, *p* = 0.021; AMD, *p* = 0.080) and 24 h (No AMD, *p* = 0.036; AMD, *p* = 0.041) (Fig. 6B). We quantified a panel of proteins involved in mitochondrial homeostasis and found significantly increased content of BNIP3 in No AMD RPE at 12 (*p* = 0.0204) and 24 h (*p* = 0.012) treatment (Fig. 6C). RPE from AMD donors had a significant increase in BNIP3 at 12 h (*p* = 0.044) but not 24 h (*p* = 0.066). There were no significant changes in Nix, MFN2, or PINK1 (Fig. 6D–F). As with FCCP experiments, CQ was included to assess autophagy flux (Supplementary Fig. 10). Control experiments with CQ did not show any significant results between RPE from No AMD and AMD donors. RPE expressing mKeima-mito were imaged after treatment with CoCl_2_ for up to 24 h. We observed a slight increase in mitophagy in both No AMD and AMD RPE after CoCl_2_ treatment (Fig. 6G + H). Individual neutral (green) and acidic (red) channels for both No AMD and AMD RPE treated with FCCP are shown (Supplementary Fig. 11). While not significantly different, AMD RPE had a larger increase in mitophagy by 24 h (~ 8%) (Fig. 6I). These results indicate that 250 µM CoCl_2_ stabilized HIF1α as expected, while leading to a mild increase in cellular autophagy and mitophagy in RPE. However, these results were similar between RPE from No AMD and AMD donors.

## Discussion

In this study, we investigated mitochondrial fission, fusion, and mitophagy in human primary RPE cultures to determine if defects in these pathways lead to the accumulation of damaged and dysfunctional mitochondria associated with AMD (Fig. 1). To investigate these pathways of mitochondrial homeostasis, treatments of FCCP and CoCl_2_ were used to stimulate mitophagy via rapid membrane depolarization and hypoxia simulation, respectively. Our results show an AMD-specific response to FCCP-induced stress in fission, fusion, and mitophagy proteins. Additionally, content of PINK1 was significantly lower in RPE from AMD donors under basal conditions. After treatment with CoCl_2_, RPE from both No AMD and AMD donors had similar increases in autophagy and mitophagy.

Previous investigations of mitochondrial morphology in RPE tissue samples and primary cultures found decreased mitochondrial surface area, indicative of mitochondrial fragmentation^9,15^. Additionally, a recent study reported decreased mitochondrial volume in AMD donor tissue samples^33^. In support of these results, we observed changes in the fission and fusion pathways that may lead to increased mitochondrial fragmentation in AMD. Of the fission proteins investigated, MFF had the most significant changes in response to FCCP-induced stress (Fig. 3D). Notably, the change in MFF content was in opposing directions for No AMD and AMD RPE, with No AMD RPE decreasing content and AMD RPE increasing MFF content in response to stress. Previous investigation of MFF overexpression found that increased MFF content led to an increase in mitochondrial fission^34^. In contrast, MFF mutants with decreased MFF activity have been associated with a reduction of fission^34^. These results suggest that the increase in MFF content observed in AMD RPE could lead to increased mitochondrial fission.

Additionally, treatment with FCCP led to a significant decrease in all three mitochondrial fusion proteins in both No AMD and AMD RPE (Fig. 4). Decreasing content of fusion proteins is a mechanism utilized by cells under stress to prevent mitochondrial fusion. MFN1 and MFN2 content, as well as OPA1 cleavage, occurs when the mitochondrial membrane potential decreases to prevent fusion, and the mitochondria attempts to undergo either repair or mitophagy^26^. While the RPE from No AMD and AMD donors had similar directional responses in all three fusion proteins investigated, there was an overall decreased content in AMD RPE. Additionally, AMD RPE were consistently delayed in their recovery in the fusion proteins investigated. These results may indicate that AMD RPE have an increased response to stress, leading to increased fragmentation and a delayed ability to undergo fusion. These data are consistent with previous reports of decreased mitochondrial surface area and altered mitochondrial morphology in AMD RPE tissue, which are isolated from a diseased environment where mitochondria may be under stress^9^.

Previous studies utilizing starvation and oxidative stress found decreased autophagy in RPE of AMD donors that may prevent adequate removal of damaged mitochondria^15,35^. While these studies provided an assessment of cellular autophagy, our study is the first to investigate mitophagy in human primary RPE from donors with AMD. We observed significant differences in mitophagy pathways at baseline and after FCCP treatment (Fig. 5). Under basal conditions, we observed an approximate 50% decrease in PINK1 content in RPE from AMD donors (Fig. 5B). Numerous studies using a human neuroblastoma cell line report that decreased PINK1 content led to increased mitochondrial fragmentation, and cell death^36–38^. Decreased PINK1 content in the RPE from AMD donors may contribute to increased mitochondrial fragmentation and loss of RPE observed in past experiments using human RPE tissue and the fission and fusion experiments conducted in this report.

After FCCP treatment, PINK1 content was similar in RPE from No AMD and AMD donors but content of Parkin was significantly lower in AMD RPE. This decrease in Parkin content following FCCP treatment may stem from lower PINK1 content under basal conditions, as PINK1 is a sensor of mitochondrial depolarization and activator of Parkin^26,39^. In addition to differences in ubiquitin-mediated mitophagy, receptor-mediated proteins were significantly decreased in RPE from AMD donors following FCCP-induced stress (Fig. 5E). While not significant, when quantifying mitophagy via mKeima-mito, we observed consistently decreased mitophagy over time in the AMD RPE. Together, these results suggest a decreased capacity for AMD RPE to induce and conduct mitophagy in response to FCCP. The increased fission response and delayed fusion response of AMD RPE, combined with these findings of reduced mitophagy, could lead to the accumulation of damaged mitochondria in AMD.

Due to the significant differences in both ubiquitin-mediated and receptor-mediated mitophagy proteins after FCCP induced stress, we further investigated these pathways by using CoCl_2_. Treatment with CoCl_2_ stabilizes HIF1α, inducing the hypoxia response, which activates receptor-mediated mitophagy^32^. While not significant, we observed consistently increased mitophagy in RPE from AMD donors after treatment with CoCl_2_. Previous reports of PINK1 or PARKIN knockouts have shown that receptor-mediated pathways are able to compensate in order to maintain mitophagy^36,40^. Our results after CoCl_2_ induced stress, combined with the significant decrease in basal PINK1 and decreased PARKIN content after FCCP treatment, suggest that AMD RPE may utilize receptor-mediated mitophagy as a compensatory mechanism for deficits in the ubiquitin-mediated mitophagy pathway.

One limitation of our study is the use of whole cell lysates to investigate mitochondrial proteins. Many mitochondrial proteins are encoded by the nuclear DNA and imported into the mitochondria, and therefore dependent on localization to the mitochondria to be active. By using whole cell lysates, we are unable to determine if a change in protein content is reflective of a change in mitochondrial localization and activity. Protocols exist for mitochondrial isolation from cells, but this technique is not possible in primary RPE due to the limited cell numbers generated from a human donor. Future studies investigating protein localization and activation could further utilize immunofluorescent labeling and mitochondrial imaging to determine localization of these proteins. Additionally, imaging experiments using high resolution microscopy would allow for the measurement of mitochondrial length and volume, providing insight into time dependent mitochondrial fission and fusion dynamics. Another limitation is that we did not investigate biogenesis, the expansion of mitochondria via increased lipid and protein production, and mtDNA replication. Investigating biogenesis presents challenges, including the requirement of methods to deplete mitochondria in order to monitor activation of these pathways. Future studies into mitochondrial regulation and mitochondrial homeostasis are required to further understand how defects in these pathways may lead to an AMD phenotype.

There are several caveats to consider when using human primary RPE cultures, including the difficulty in acquiring samples, limited cell numbers, and donor-to-donor variability. Additionally, primary RPE are cultured from the donor eyes of individuals age 60 years or older, and in the case of an AMD donor, have been living and surviving in the diseased retina. It is possible that only the healthiest cells are able to survive our culture conditions, thereby limiting our ability to assess the full range of defects present in the RPE of AMD donors. This idea is supported by the increased resistance of AMD RPE to peroxide-induced stress, suggesting these cells have been preconditioned to handle stress^14^.

The observed response to FCCP-induced stress highlights potential defects in mitochondrial homeostasis that may contribute to the death of RPE in AMD. Previous studies of AMD RPE graded for disease severity have observed an accumulation of mitochondrial damage and dysfunction that begins early in disease^2,9–11,13,14,23,41–43^. Current models of AMD pathogenesis center on metabolic uncoupling, in which defects in RPE mitochondria lead to the starvation of photoreceptors^8,44^. Since RPE rely on photoreceptors for various nutrients, this starvation begins a cycle of escalating metabolic changes that culminates in the death of RPE and photoreceptors leading to vision loss^8^.

The central role of mitochondria in RPE health and development of AMD has led to a number of studies testing the efficacy of mitochondrial targeted drugs. For example, the tetrapeptide SS-31 targets mitochondrial cardiolipin, which is crucial for maintaining mitochondrial oxidative phosphorylation, is being investigated in a phase 2 clinical trial for dry AMD^45^. The antidiabetic drug metformin has been shown to activate AMPK, promoting mitochondrial biogenesis. A recent study of over 7000 diabetic patients taking metformin found that metformin treatment was linked to reduced risk for AMD^46^. Additional compounds designed to protect mitochondria from oxidative damage (N-acetyl-L-Cysteine; NAC), remove damaged mitochondria via increased autophagy (rapamycin), upregulate mitochondrial biogenesis (pyrroloquinoline; PQQ), or improve oxidative phosphorylation (nicotinamide mononucleotide, NMN) have been investigated in our lab^47^. We found that RPE from AMD donors responded to the drugs as detected by the increase in mitochondrial function. In contrast, RPE from donors without AMD did not respond to the drugs. These results are consistent with the idea that mitochondrial dysfunction in the diseased cells can be ameliorated by treatments that target mitochondrial defects. Our study supports the idea that mitochondrial defects drive AMD, and targeting pathways of mitochondrial homeostasis may be a viable treatment option.

This study uncovers potential mechanisms leading to mitochondrial damage and dysfunction associated with AMD that may initiate the metabolic crisis in the retina. We observed disease-specific differences under basal conditions and in response to two mitochondrial stressors, FCCP and CoCl_2_. The AMD-associated changes in mitochondrial proteins reported in this study could lead to the buildup of damaged and dysfunctional mitochondria that begins to disrupt the delicate retinal ecosystem and lead to the eventual death of RPE and photoreceptors in AMD. Experiments using different stressors and analytical methods may further identify mechanisms that explain these AMD-associated changes in mitochondrial homeostasis, providing new therapeutic targets to treat AMD.

## Supplementary Information

Supplementary Information 1.

Supplementary Information 2.

## Data Availability

All data generated or analyzed during this study are included in this published article (and its Supplementary Information files).
