# Supplementary material for: Investigating mitochondrial fission, fusion, and autophagy in retinal pigment epithelium from donors with age-related macular degeneration
Source: Sci Rep. 2022 Dec 16;12:21725. doi: 10.1038/s41598-022-26012-5 (PMC9758189; doi:10.1038/s41598-022-26012-5)
Supplement: Supplementary file 1 — Supplementary Information 1. [file 41598_2022_26012_MOESM1_ESM.docx]

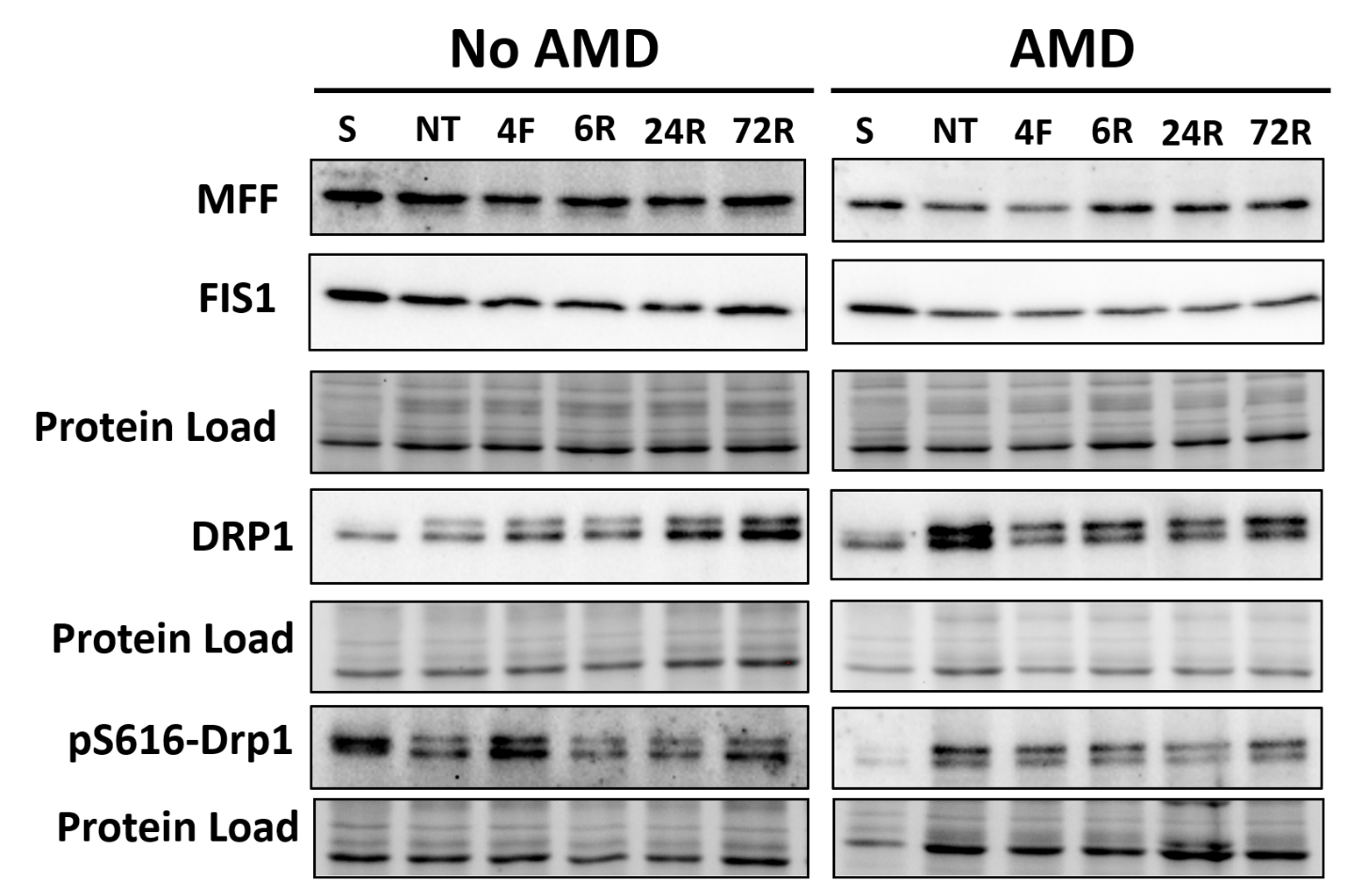


**Supplementary Figure 1. Representative blots for mitochondrial fission proteins.** Corresponds with Figure 3. Sample abbreviations: Standard (S), No Treatment Control (NT), 4 hours FCCP (4F), 6, 24, and 72 hour recovery (6R, 24R, 72R).


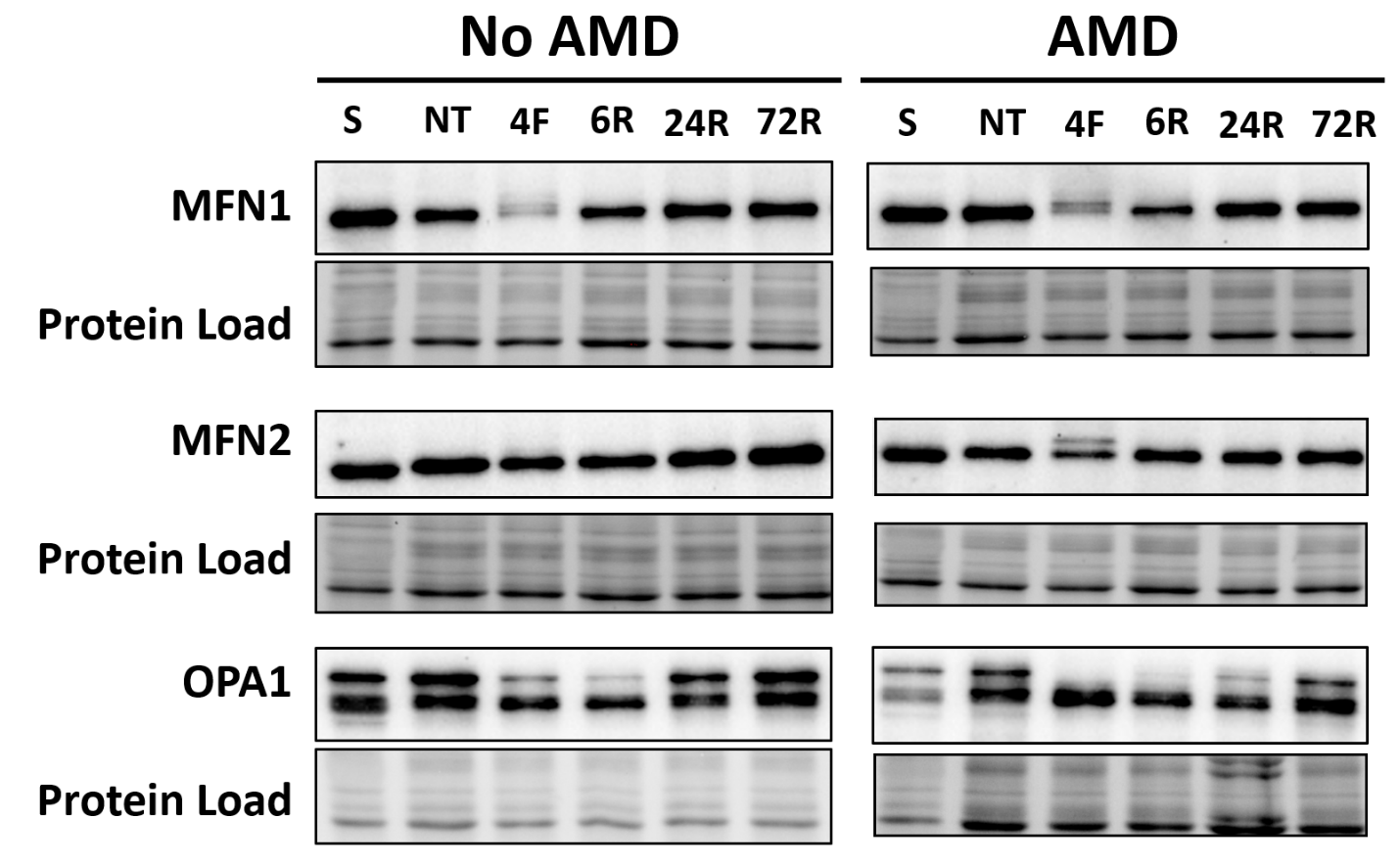


**Supplementary Figure 2. Representative blots for mitochondrial fusion proteins.** Corresponds with Figure 4. Sample abbreviations: Standard (S), No Treatment Control (NT), 4 hours FCCP (4F), 6, 24, and 72 hour recovery (6R, 24R, 72R).


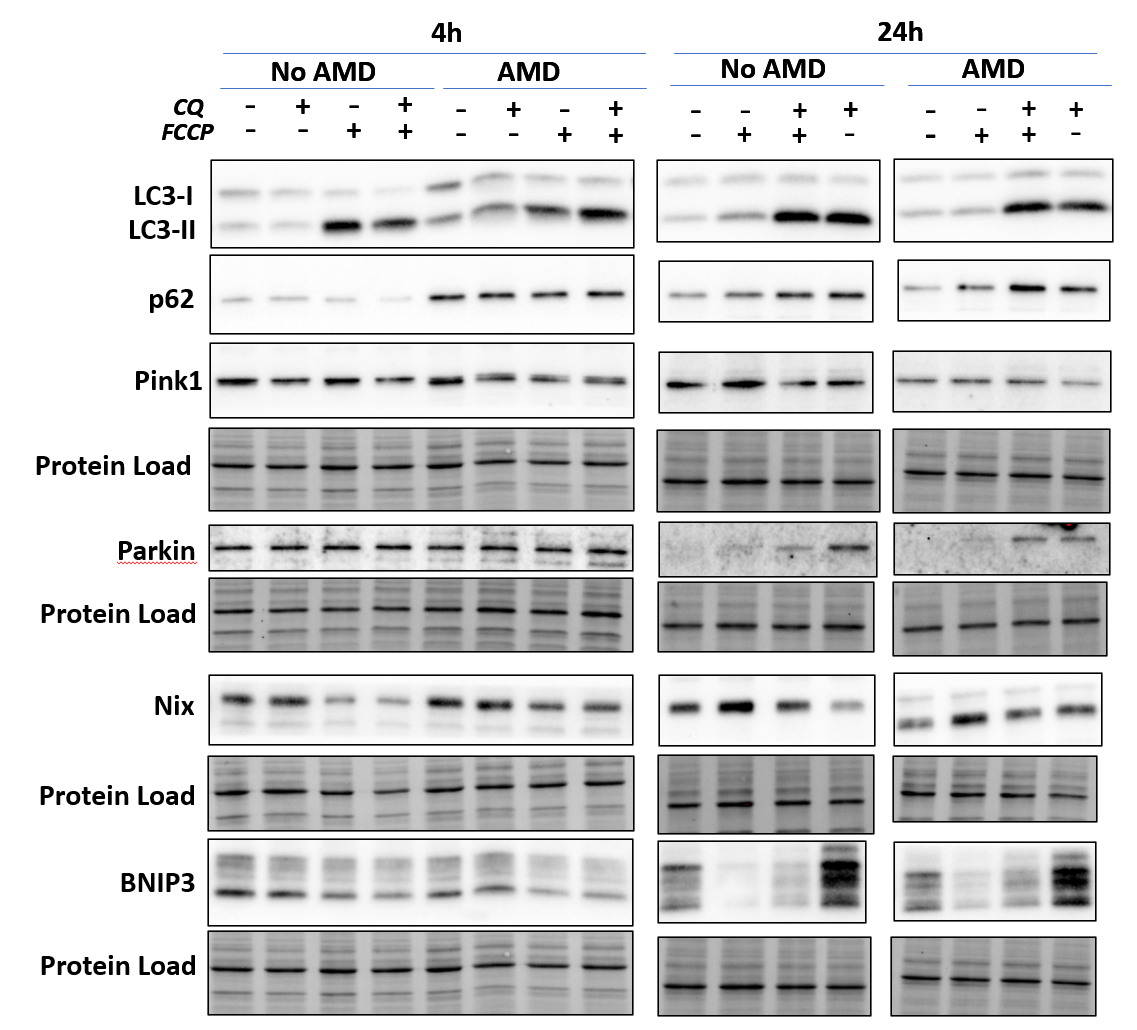


**Supplementary Figure 3. Representative blots for mitophagy proteins.** Corresponds with Figure 5 and Supplemental Figure 6.


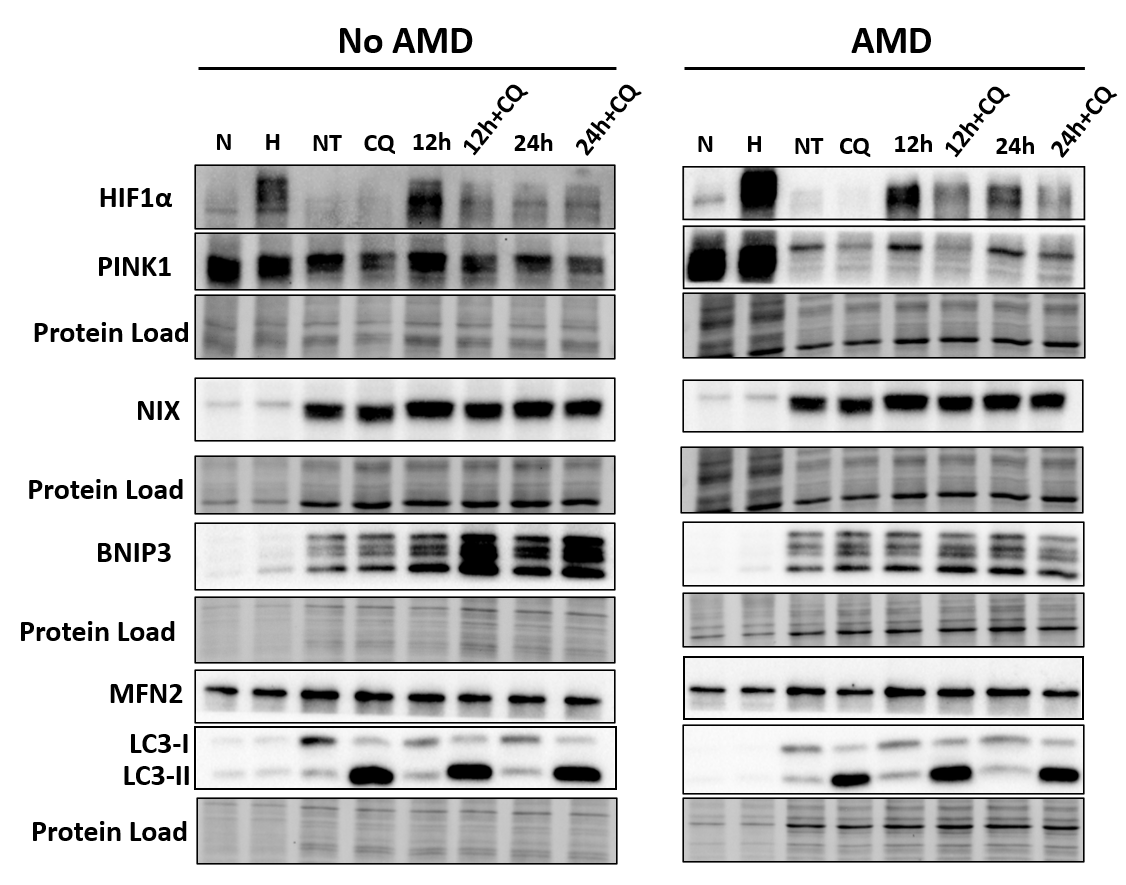


**Supplementary Figure 4. Representative blots for proteins after CoCl_2_ treatments.** Corresponds with Figure 6 and Supplemental Figure 9.


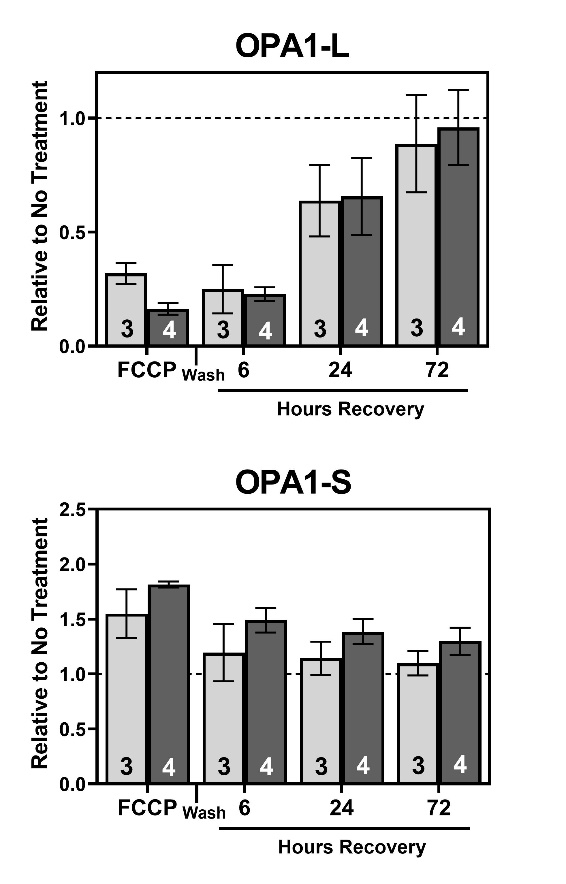


**Supplementary Figure 5. OPA1 isoforms after FCCP treatment and throughout recovery**. Quantification of OPA1-L (top) and OPA1-S (bottom) isoforms following treatment with 4 hours of FCCP and at 6, 24, and 72 hours of recovery from FCCP treatment.


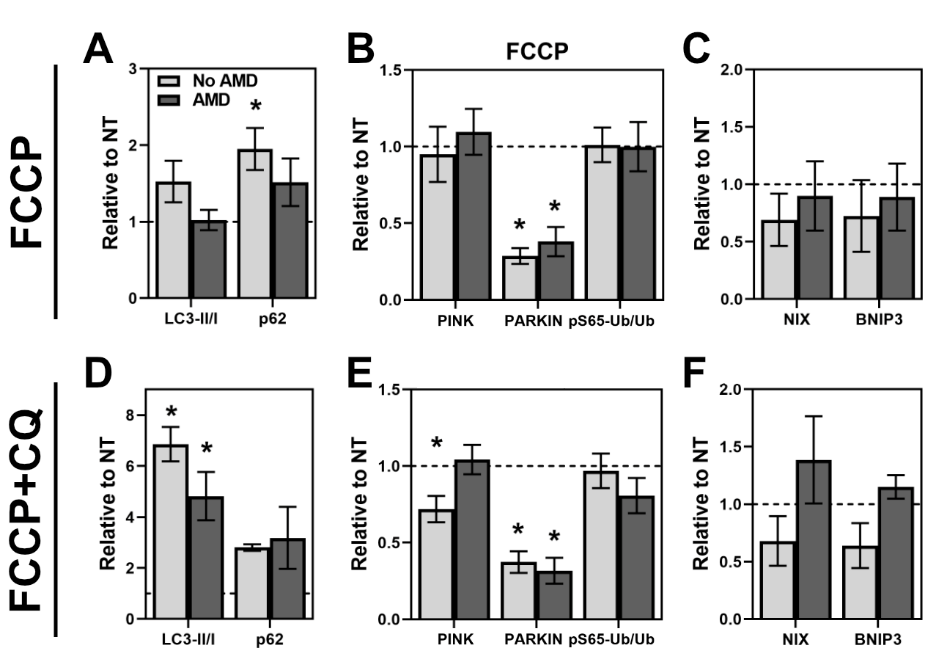


**Supplementary Figure 6. Quantification of proteins following 24 hours FCCP treatment.** (A-C) Quantification of autophagy proteins LC3-II/I and p62 (A), PINK and Parkin (B), or Nix and BNIP 3 (C) after 24 hours FCCP treatment. (D-F) Quantification of autophagy proteins LC3-II/I and p62 (D), PINK and Parkin (E), or Nix and BNIP 3 (F) after 24 hours FCCP and 2 hours CQ treatment.


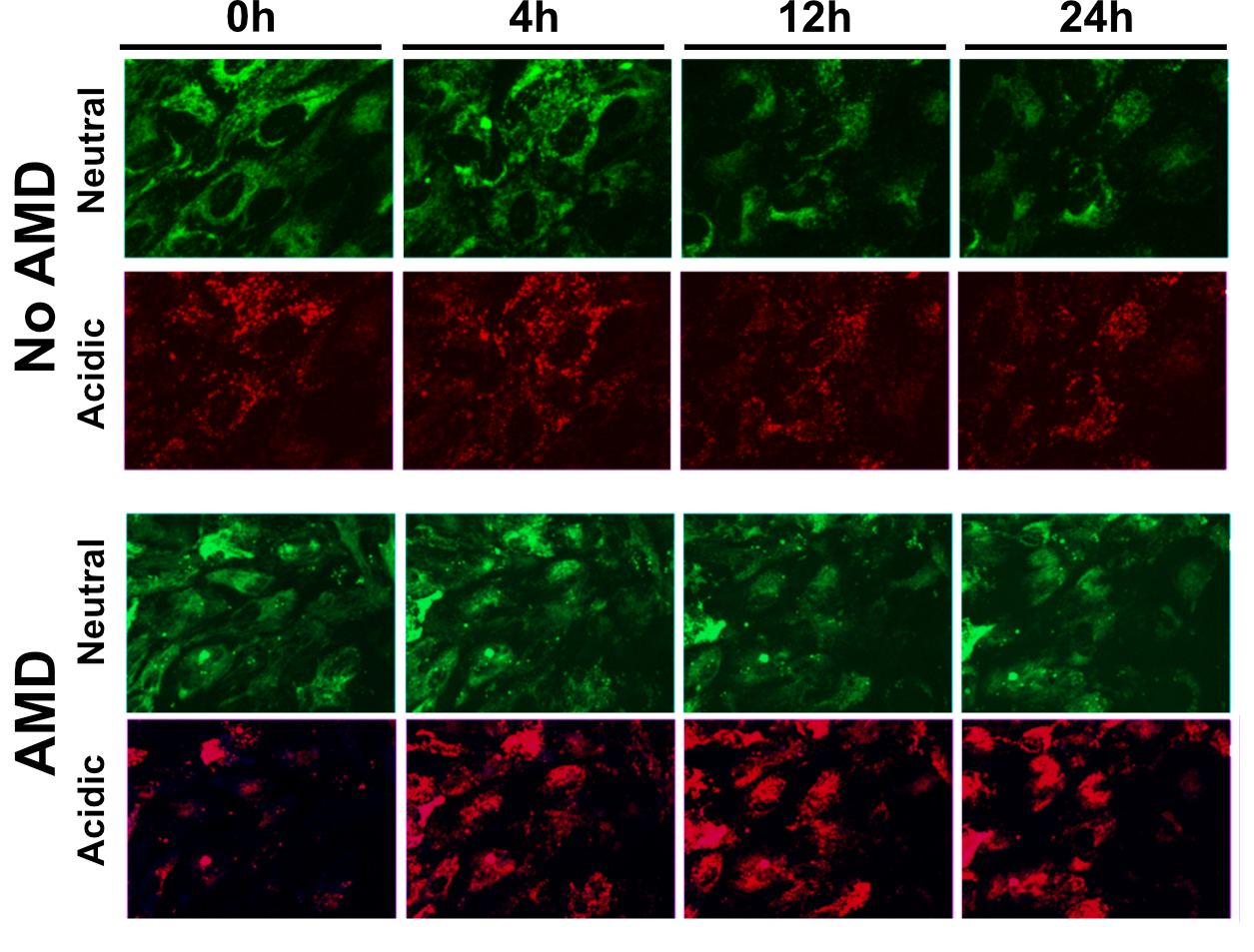


**Supplementary Figure 7. Individual neutral and acidic channels after FCCP treatment.** Representative images of mKeima-mito in primary RPE cultures from No AMD (top) and AMD (bottom) donors. Both neutral (green) and acidic (red) channels are shown. Images correspond to Figure 5 in text.


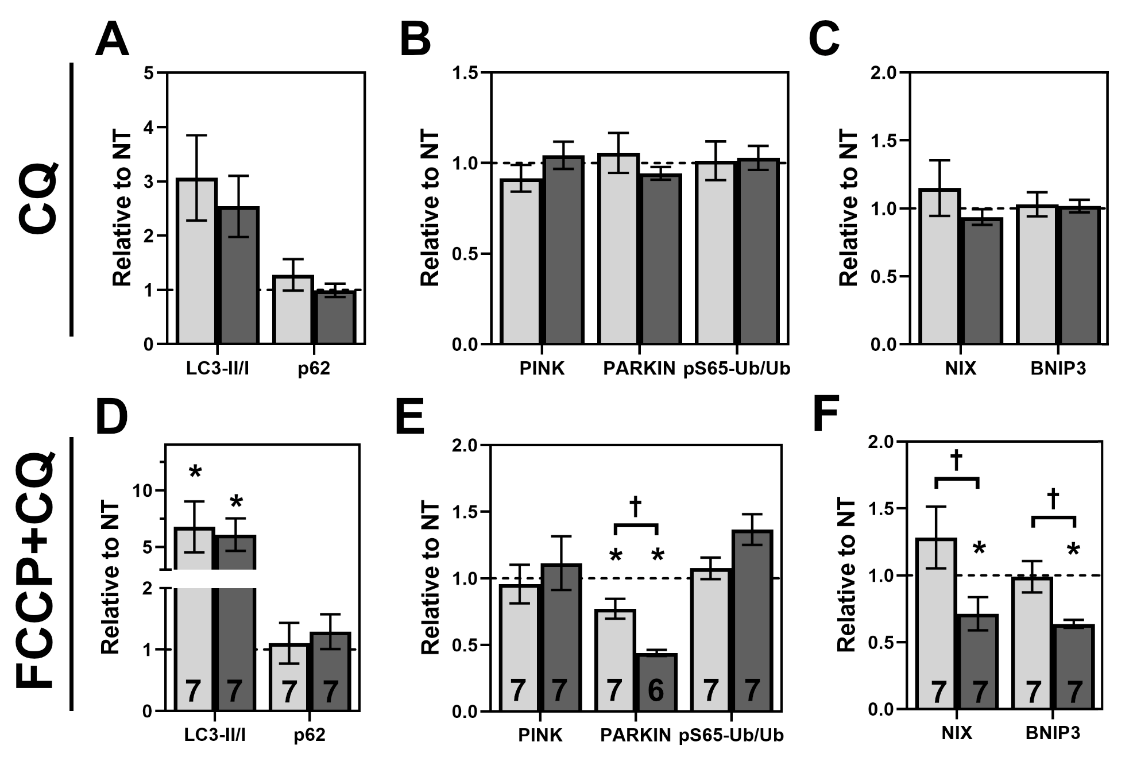


**Supplementary Figure 8. Quantification of mitophagy proteins after CQ treatment.** (A-C) Content of LC3-II/I and p62 (A), PINK, Parkin, and pS65-Ub/Ub (B), or Nix and BNIP3 (C) following 2 hours CQ treatment. (D-F) Content of LC3-II/I and p62 (D), PINK, Parkin, and pS65-Ub/Ub (E), or Nix and BNIP3 (F) following 4 hours FCCP with 2 hours CQ treatment.


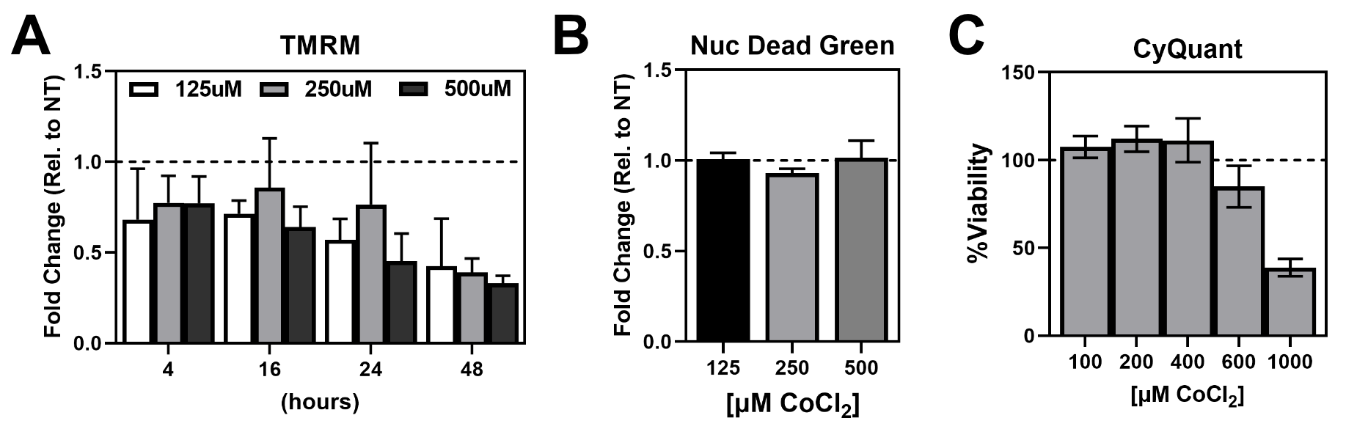


**Supplementary Figure 9. Characterization of Cobalt Chloride.** (A) Mitochondrial membrane potential relative to untreated controls for 125, 250, and 500μM CoCl_2_ across multiple time points (x-axis) (n=3). (B) Quantification of Nuc-Dead Green (Thermo Fisher) after 24 hours of CoCl_2_ (n=3). (C) Viability measured with CyQuant after 24 hours treatment with multiple doses of CoCl_2_ (x-axis) (n-2).


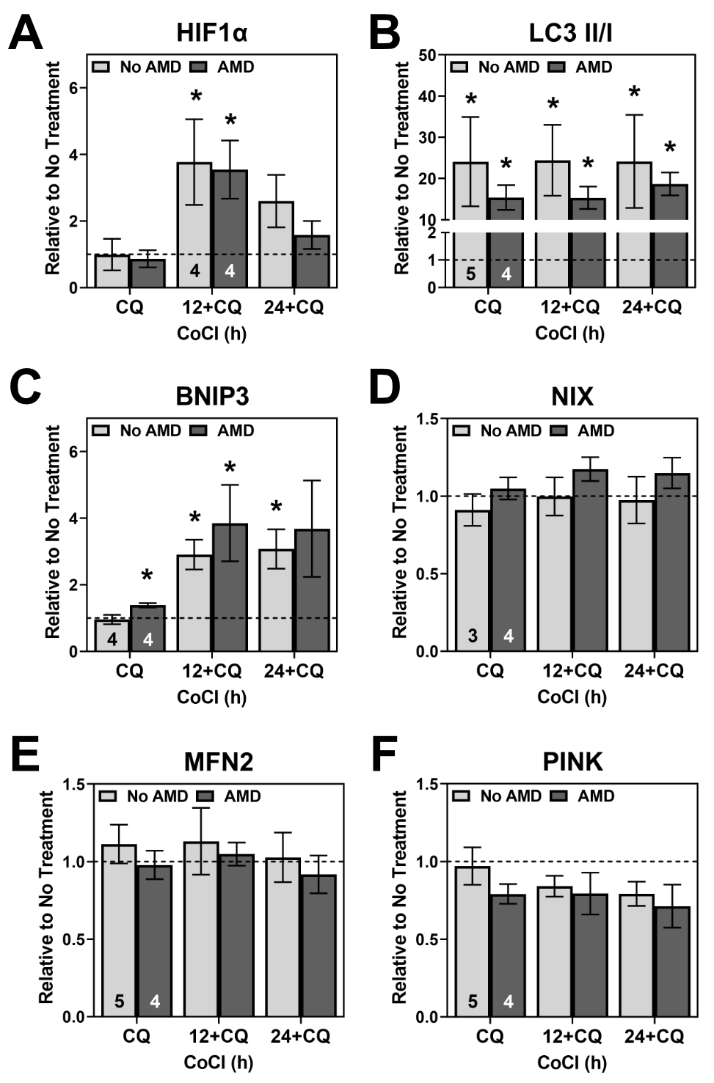


**Supplementary Figure 10. Panel of mitochondrial homeostasis proteins after cobalt chloride and chloroquine treatment.** (A-F) Quantification of proteins after treatment with 2 hours CQ, 12 hours CoCl_2_ and 2 hours CQ, or 24 hours CoCl_2_ and 2 hours CQ.


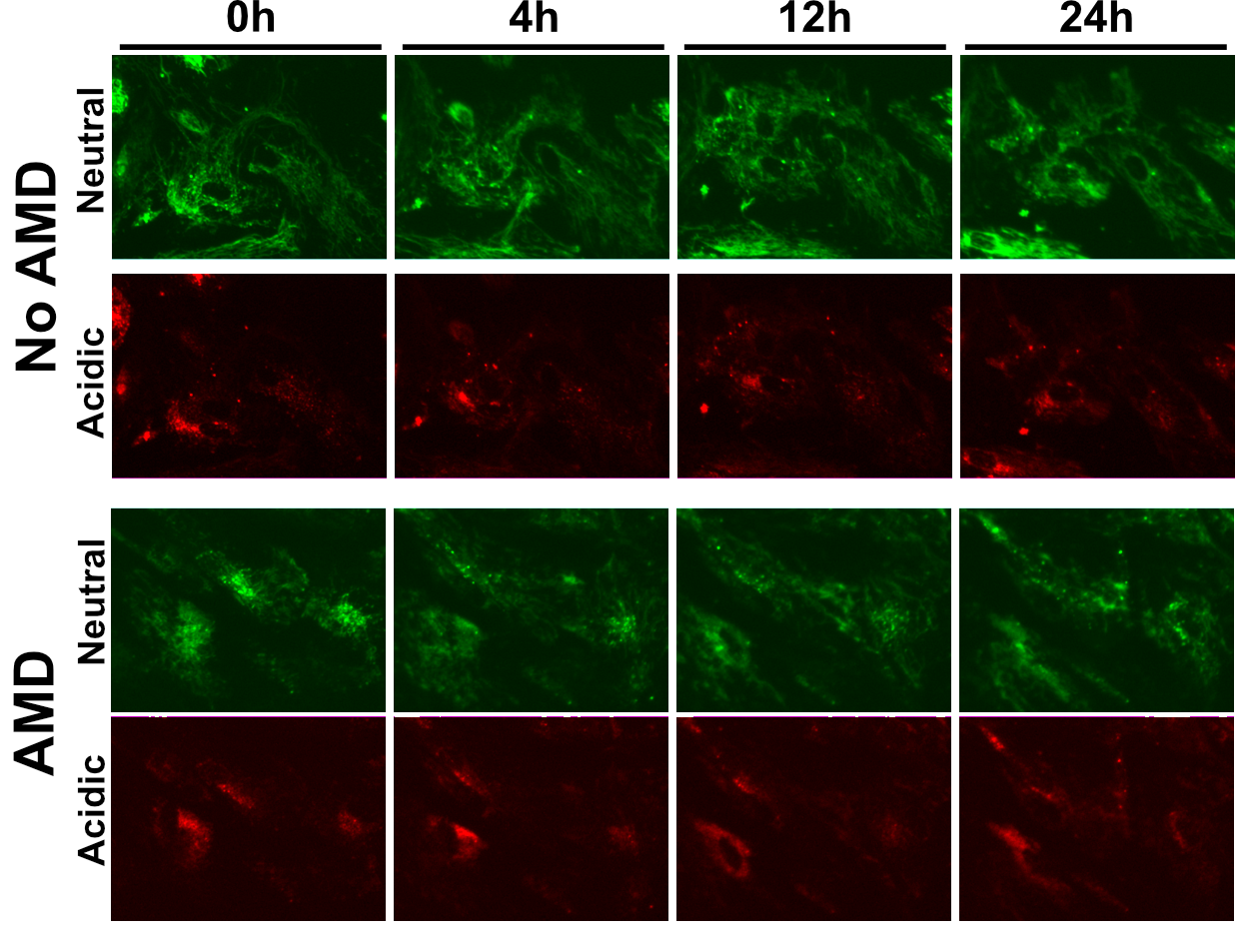


**Supplementary Figure 11. Individual neutral and acidic channels after CoCl_2_ treatment.** Representative images of mKeima-mito in primary RPE cultures from No AMD (top) and AMD (bottom) donors. Both neutral (green) and acidic (red) channels are shown. Images correspond to Figure 6 in text.

**Supplementary Table S1.** Donor demographics ^A^

| **Disease**  **State** ^B^ | **Sample** ^C^  **(n)** | **Sex**  **Male (n)** | **Sex**  **Female (n)** | **Age**  **(Mean ± SD)** | **Cause of Death** ^D^ **(n)** |
| --- | --- | --- | --- | --- | --- |
| **No AMD RPE** | 14 | 8 | 6 | 69±9 | Cancer (4), Dementia (1), Heart Failure (3), PE (1), Exsanguination (1), Sepsis (3), COPD (1) |
| **AMD RPE** | 19 | 13 | 6 | 76±8 | Cancer (3), Heart Failure (3), PE (1), Sepsis (3), COPD (1), ABI (2), Natural (1), Respiratory Failure (1), Brain Aneurysm (1), ALS (1), CVA (1), interstitial lung disease (1), |
|  |  |  |  |  |  |

ABI = anoxic brain injury; ALS = amyotrophic lateral sclerosis; CVA = cerebrovascular accident (stroke); PE = pulmonary embolism.

^A^ Information supplied by Lions Gift of Sight, St. Paul, MN.

^B^ Minnesota Grading System (MGS) was used to evaluate the stage of AMD in eye bank eyes^23^. No AMD = MGS1; AMD = MGS2 and MGS3.

^C^ Sample number indicates the total donors with or without AMD used in the current study.

^D^ The number of donors for each cause of death is indicated in parentheses.

**Supplementary Table 2: Antibodies used for Western immunoblotting.**

| **Antibody** | **Company** | **Product number** | **Dilution** |
| --- | --- | --- | --- |
| MFN1 | Cell Signaling | 14739S | 1:1000 |
| MFN2 | Cell Signaling | 9482S | 1:1000 |
| OPA1 | Cell Signaling | 80471S | 1:1000 |
| FIS1 | abcam | 156865 | 1:1000 |
| DRP1 | Cell Signaling | 8570S | 1:1000 |
| pDrp1-S616 | Cell Signaling | 3455S | 1:1000 |
| MFF | abcam | ab81127 | 1:1000 |
| LC3A/B | Cell Signaling | 4108S | 1:1000 |
| PINK1 | Cell Signaling | 6946S | 1:1000 |
| PARKIN | Cell Signaling | 4211S | 1:1000 |
| BINP3 | Cell Signaling | 44060S | 1:1000 |
| NIX | Cell Signaling | 12396S | 1:1000 |
| FUNDC1 | abcam | 224722 | 1:1000 |
| P62 | abcam | 109012 | 1:1000 |
| Ubiquitin | Cell Signaling | 3933S | 1:1000 |
| pS65-Ubiquitin | Boston Biochem | A-110-050_10068318 | 1:1000 |
| HIF1α | Novus | NBP2-75977 | 1:1000 |
| Anti-mouse HRP-linked secondary antibody | Cell Signaling | 7076S | 1:2000 |
| Anti-rabbit HRP-linked secondary antibody | Cell Signaling | 7074S | 1:2000 |
