# Supplementary material for: Investigating mitochondrial fission, fusion, and autophagy in retinal pigment epithelium from donors with age-related macular degeneration
Source: Sci Rep. 2022 Dec 16;12:21725. doi: 10.1038/s41598-022-26012-5 (PMC9758189; doi:10.1038/s41598-022-26012-5)

Supplementary Figure 1.

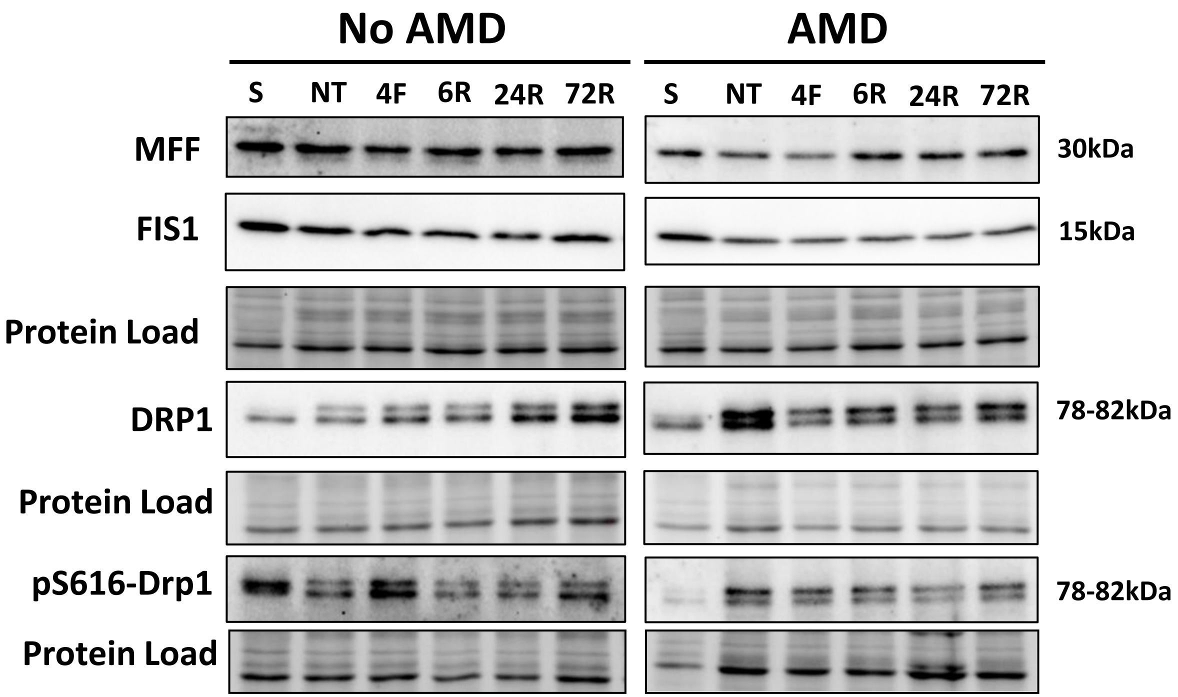

Supplementary Figure 1.

Denotes corresponding region shown in Supp. Figure.

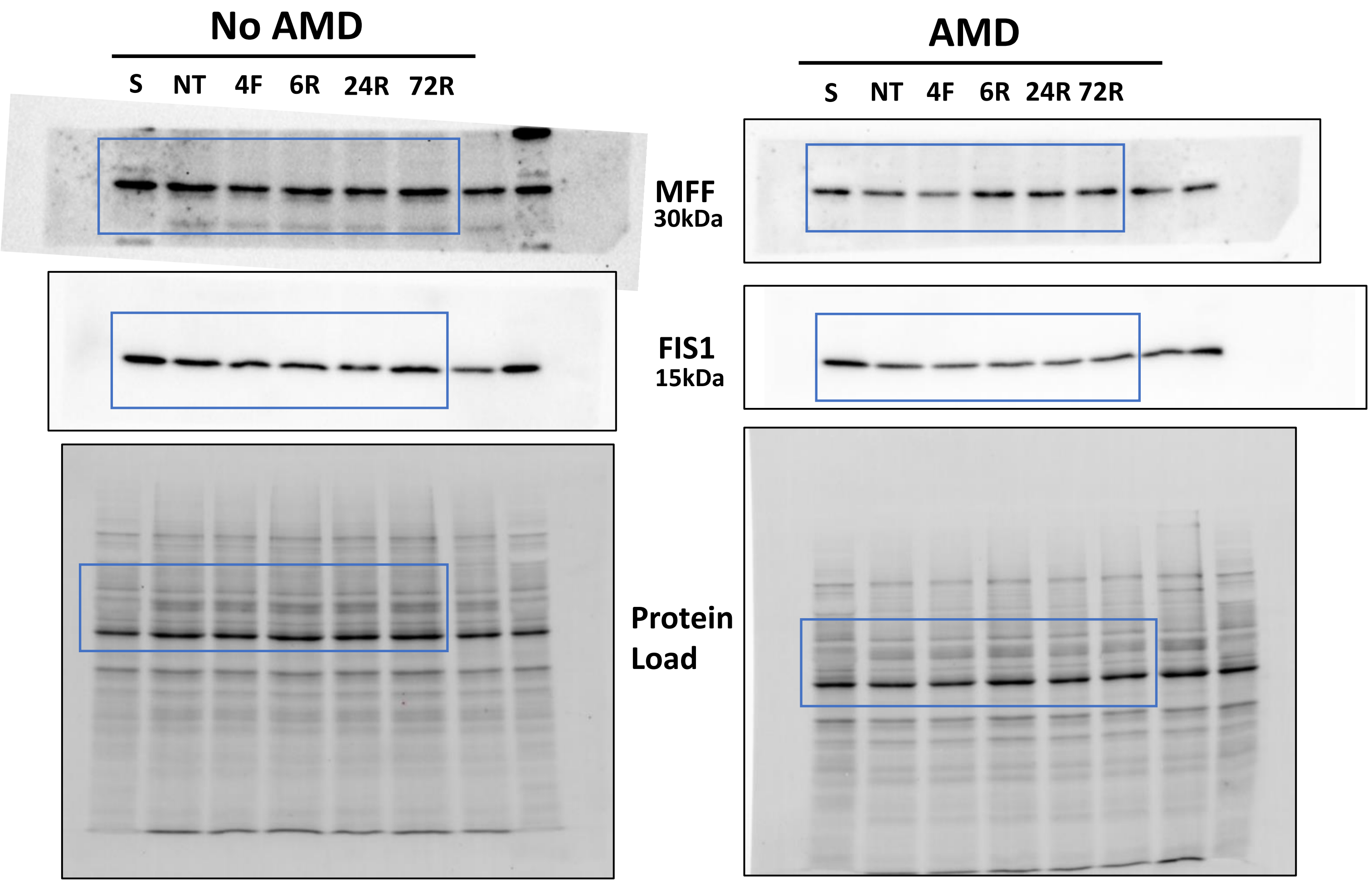

Supplementary Figure 1.

Denotes corresponding region shown in Supp. Figure.

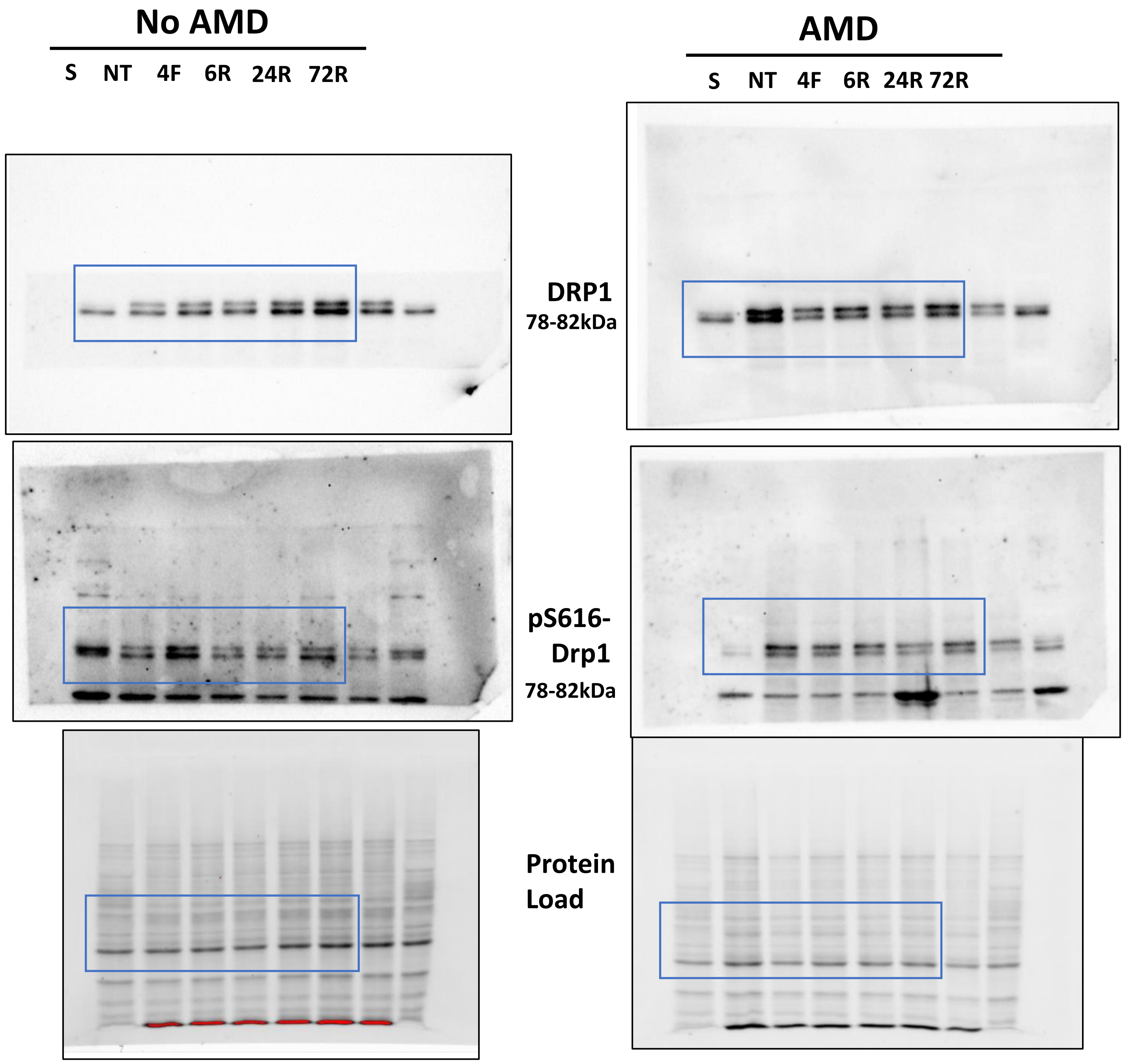

Supplementary Figure 2

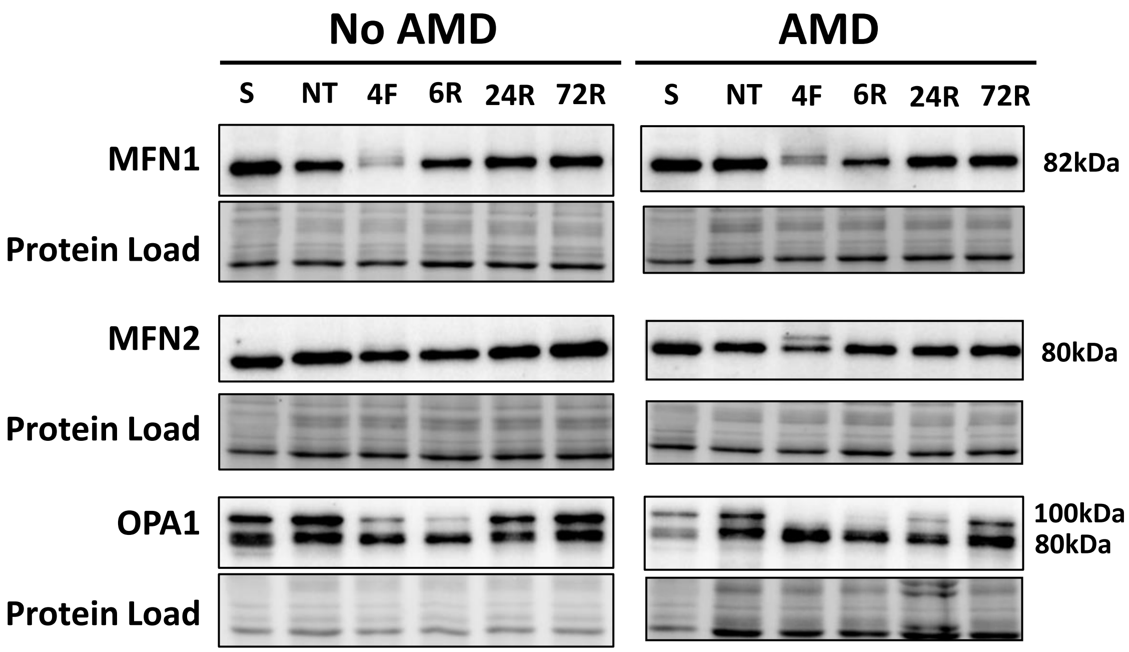

Supplementary Figure 2.

Denotes corresponding region shown in Supp. Figure.

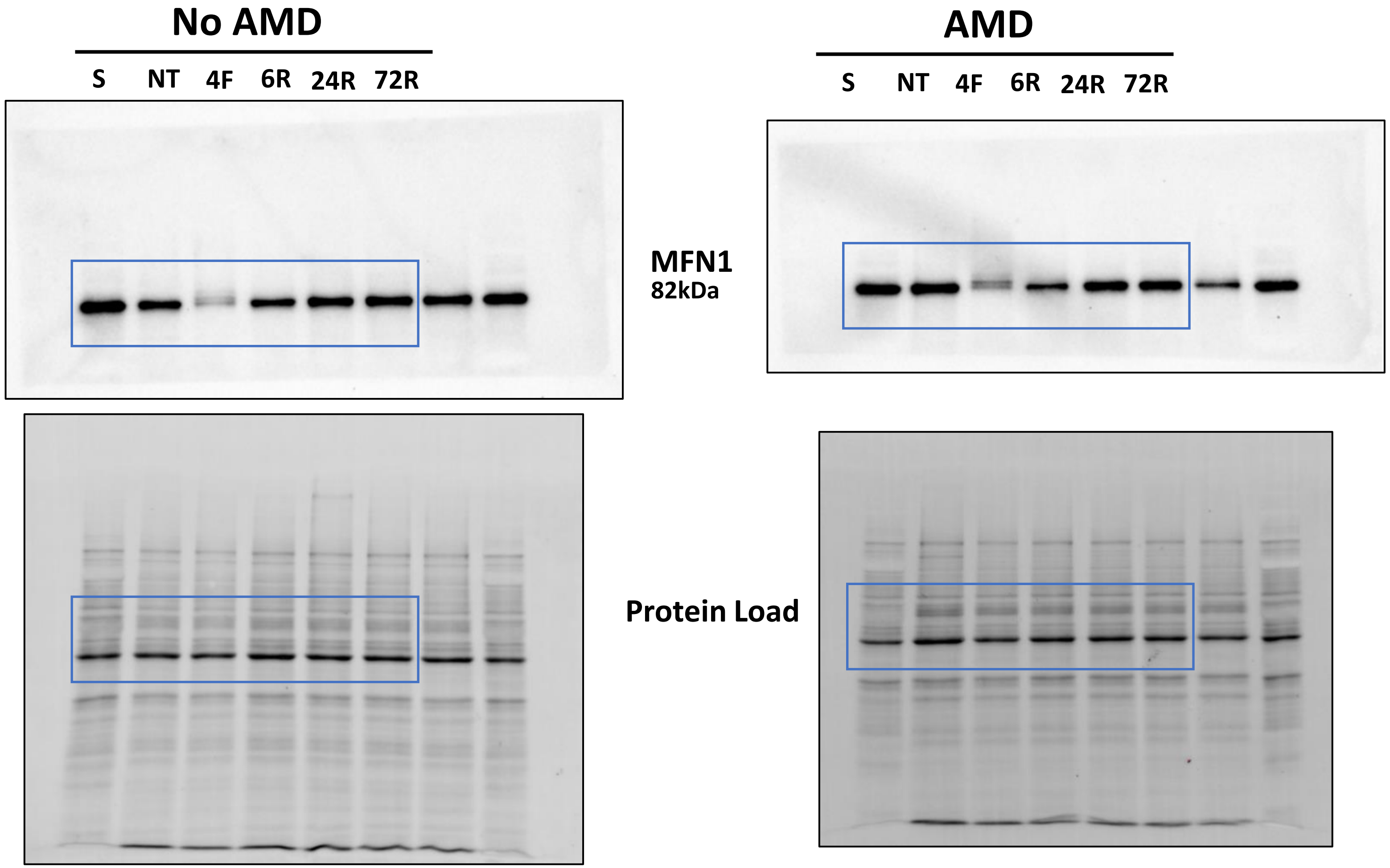

Supplementary Figure 2.

Denotes corresponding region shown in Supp. Figure.

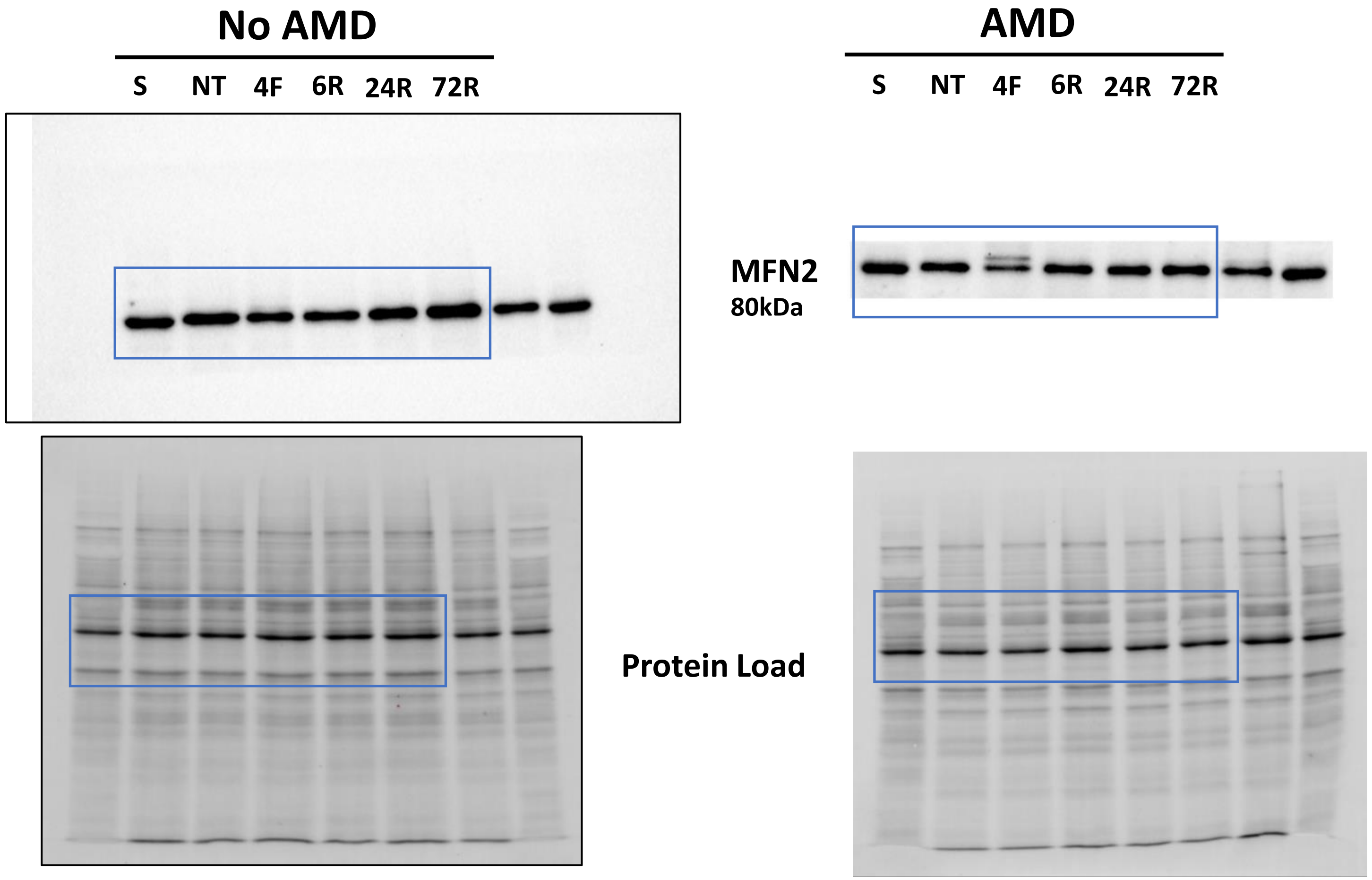

Supplementary Figure 2.

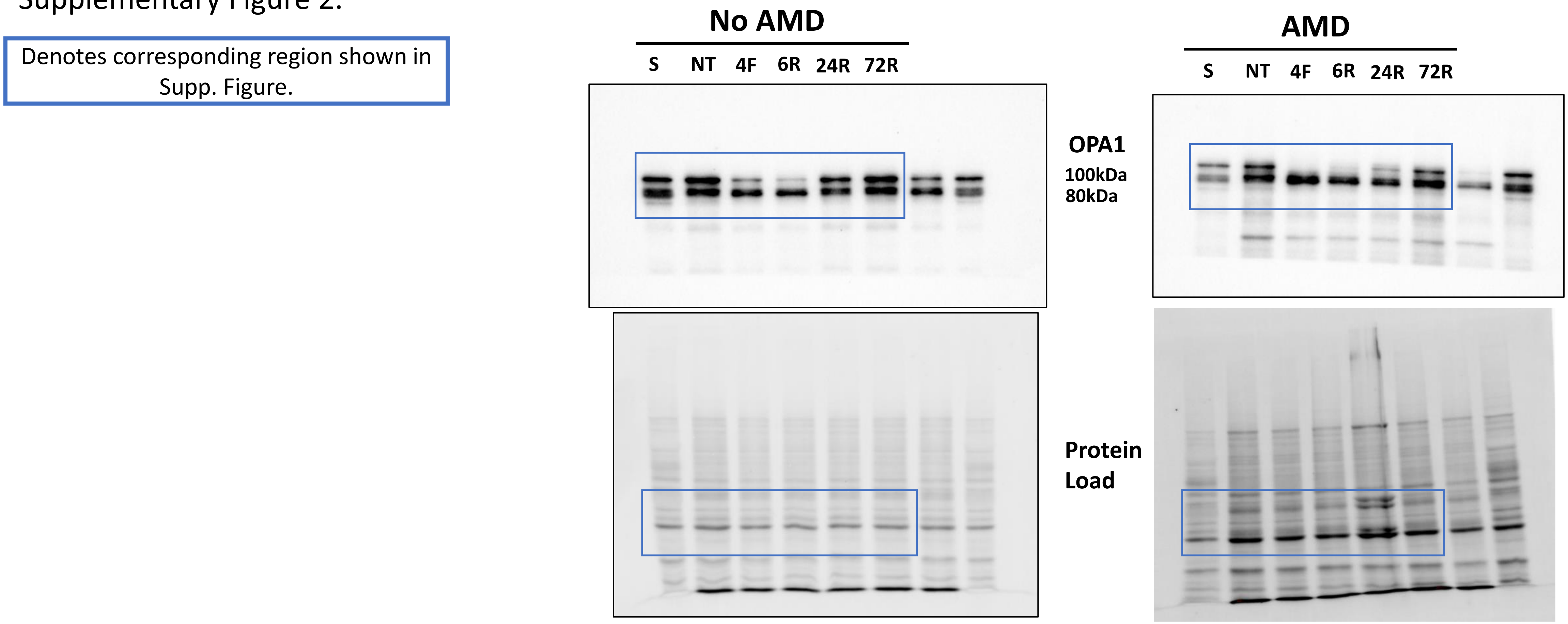

Supplementary Figure 3.

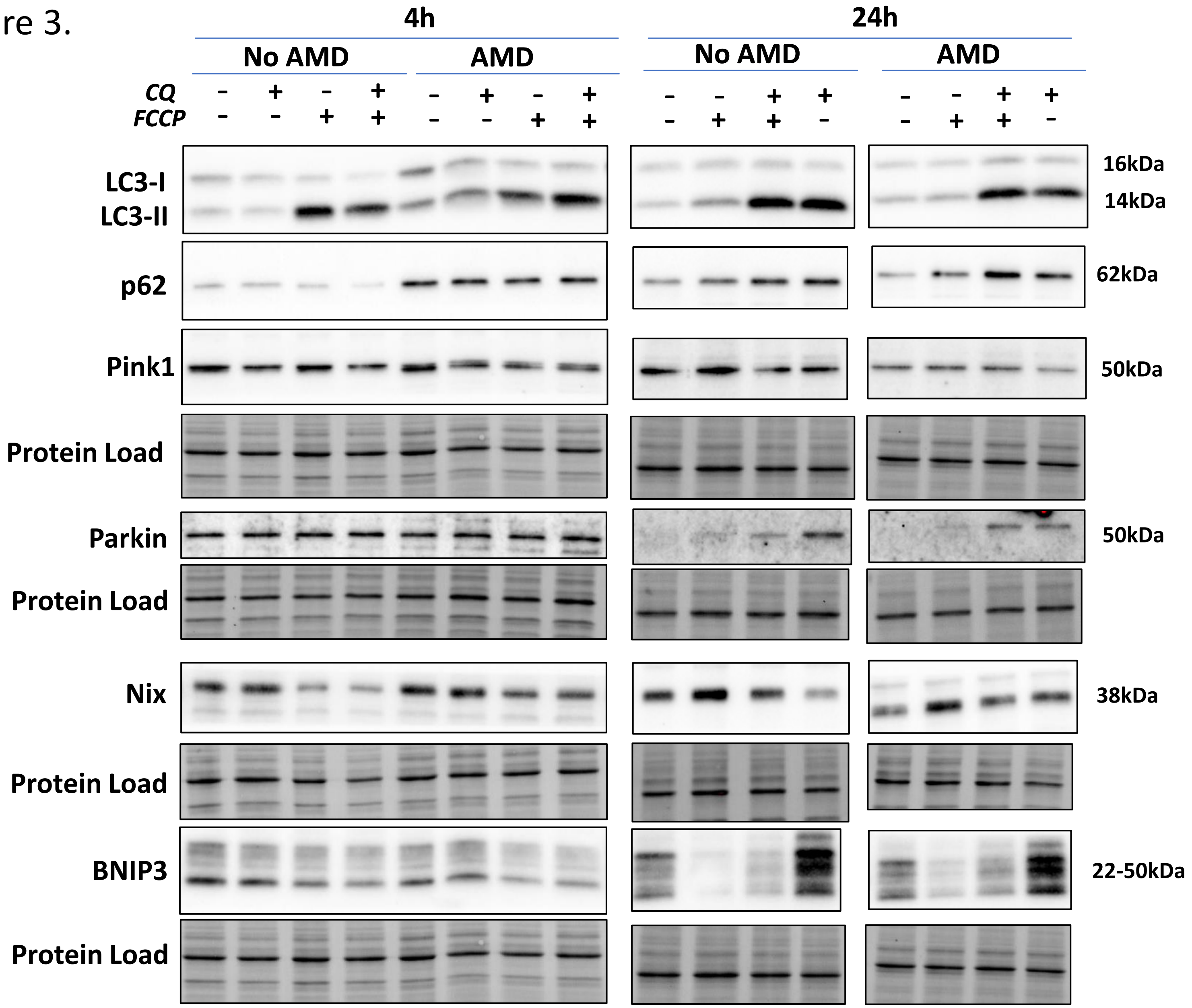

Supplementary Figure 3.

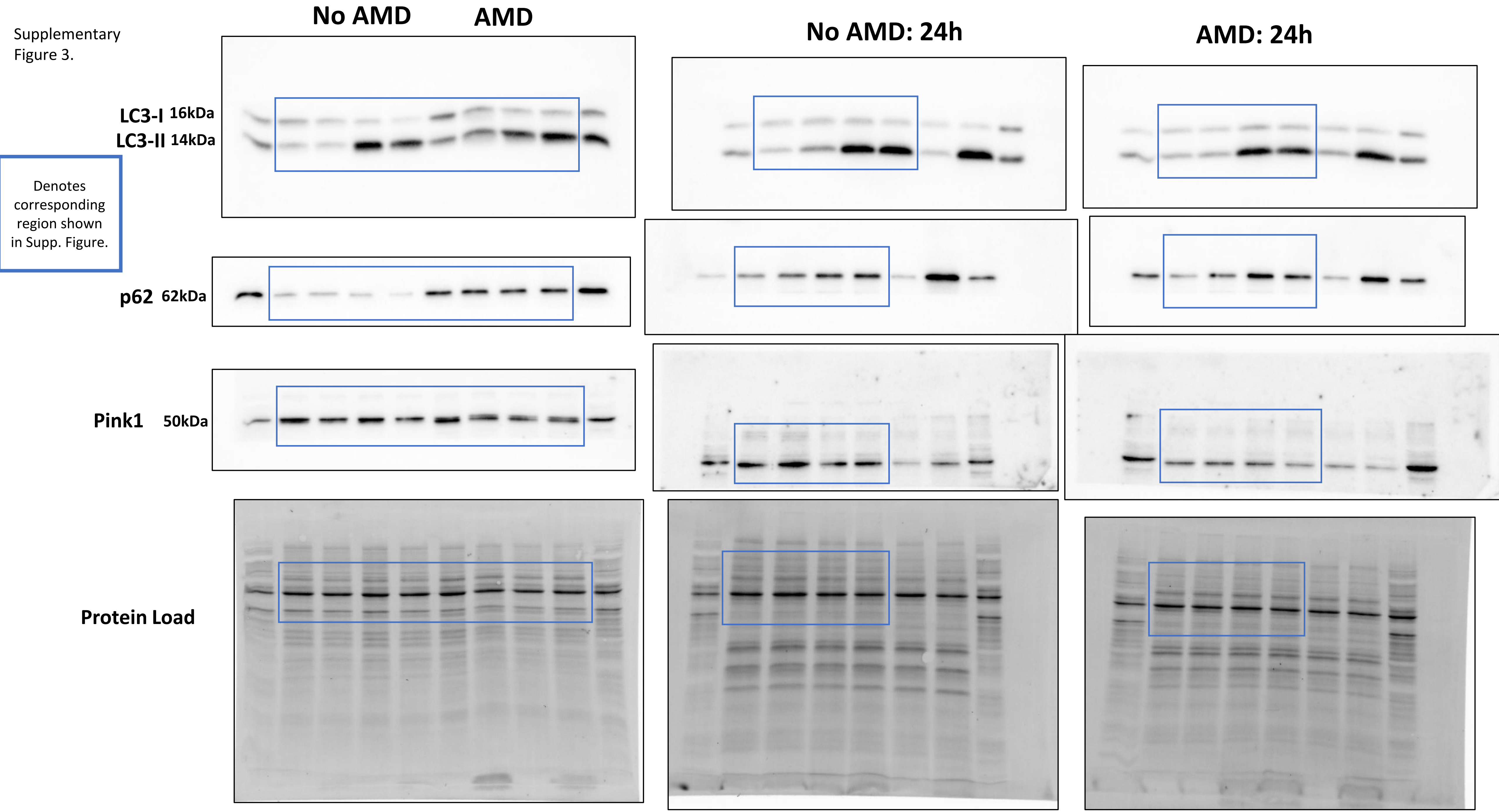

Supplementary Figure 3.

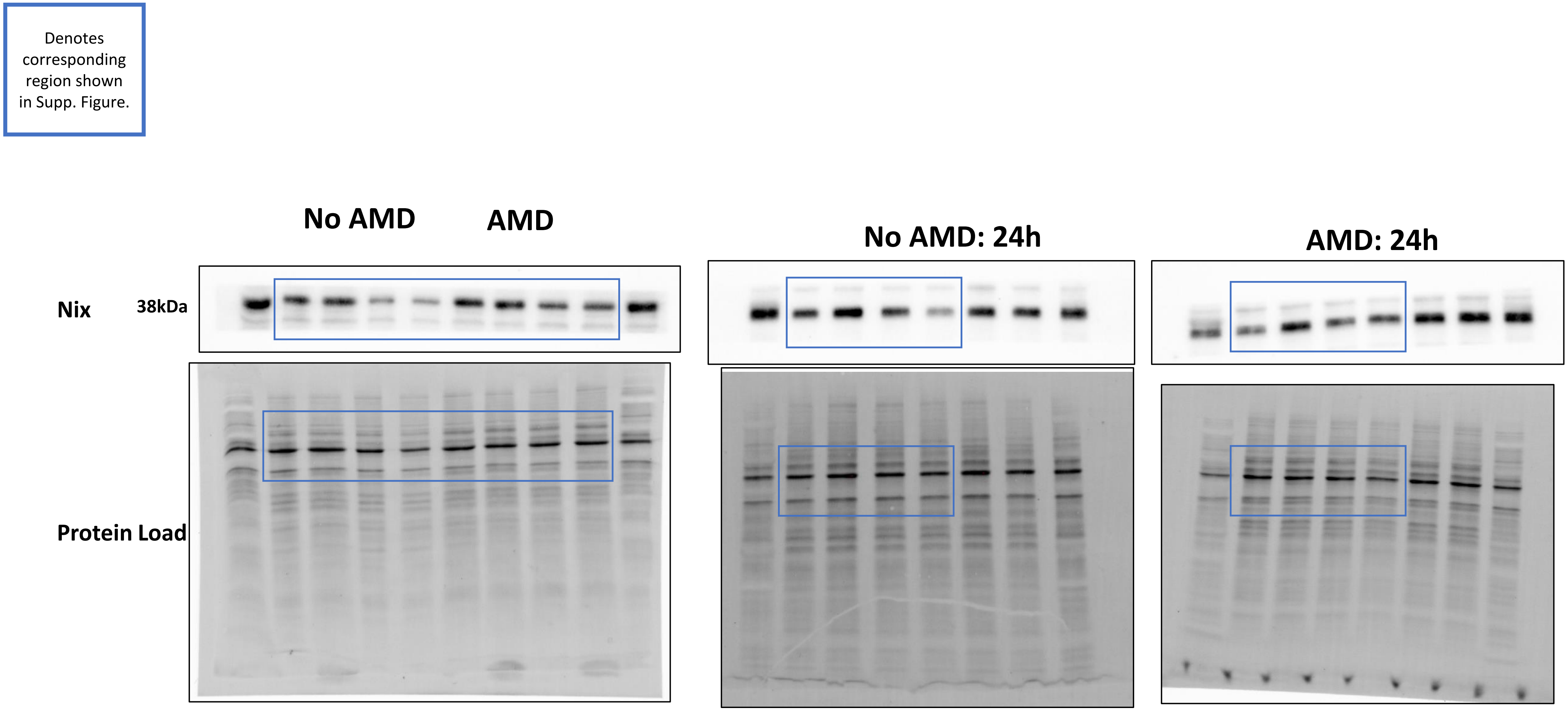

Supplementary Figure 3.

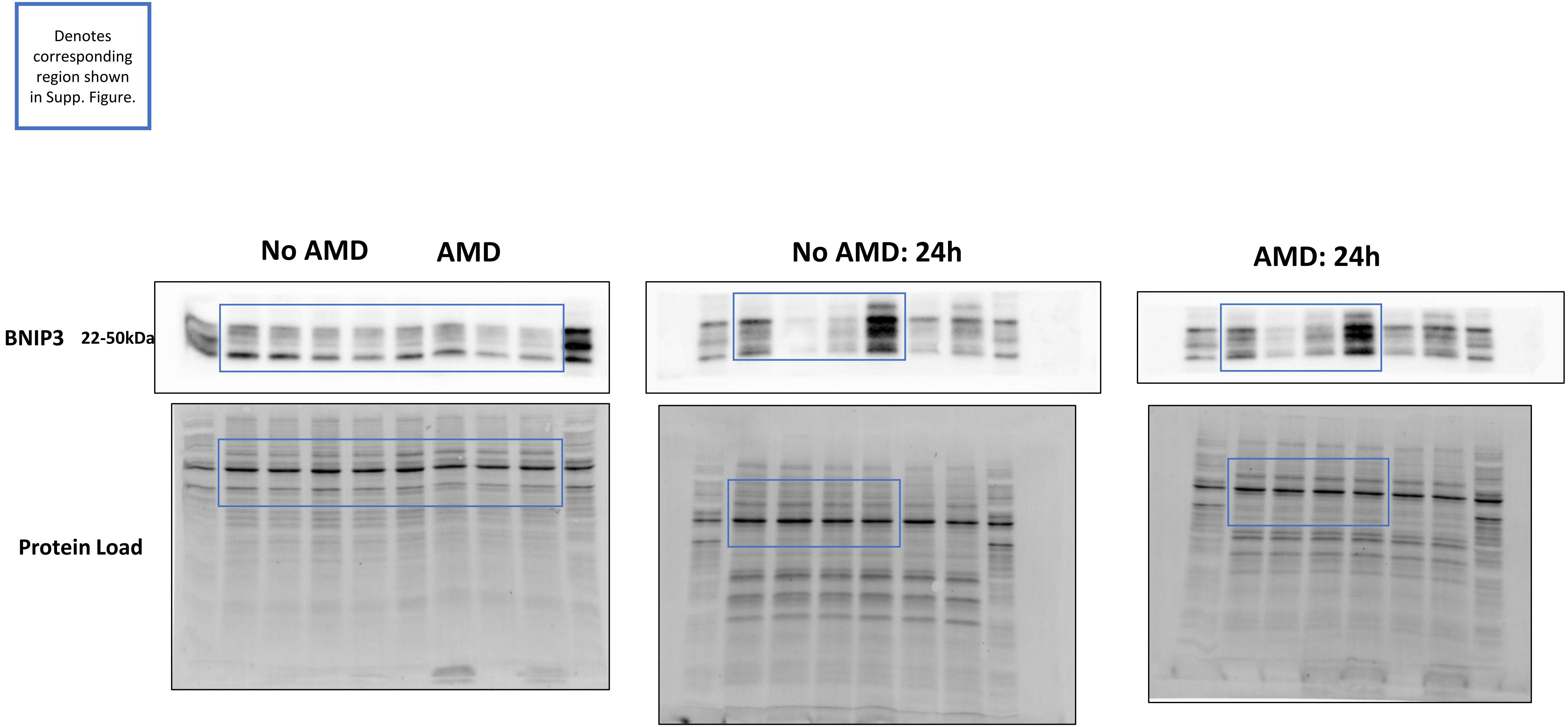

Supplementary Figure 4.

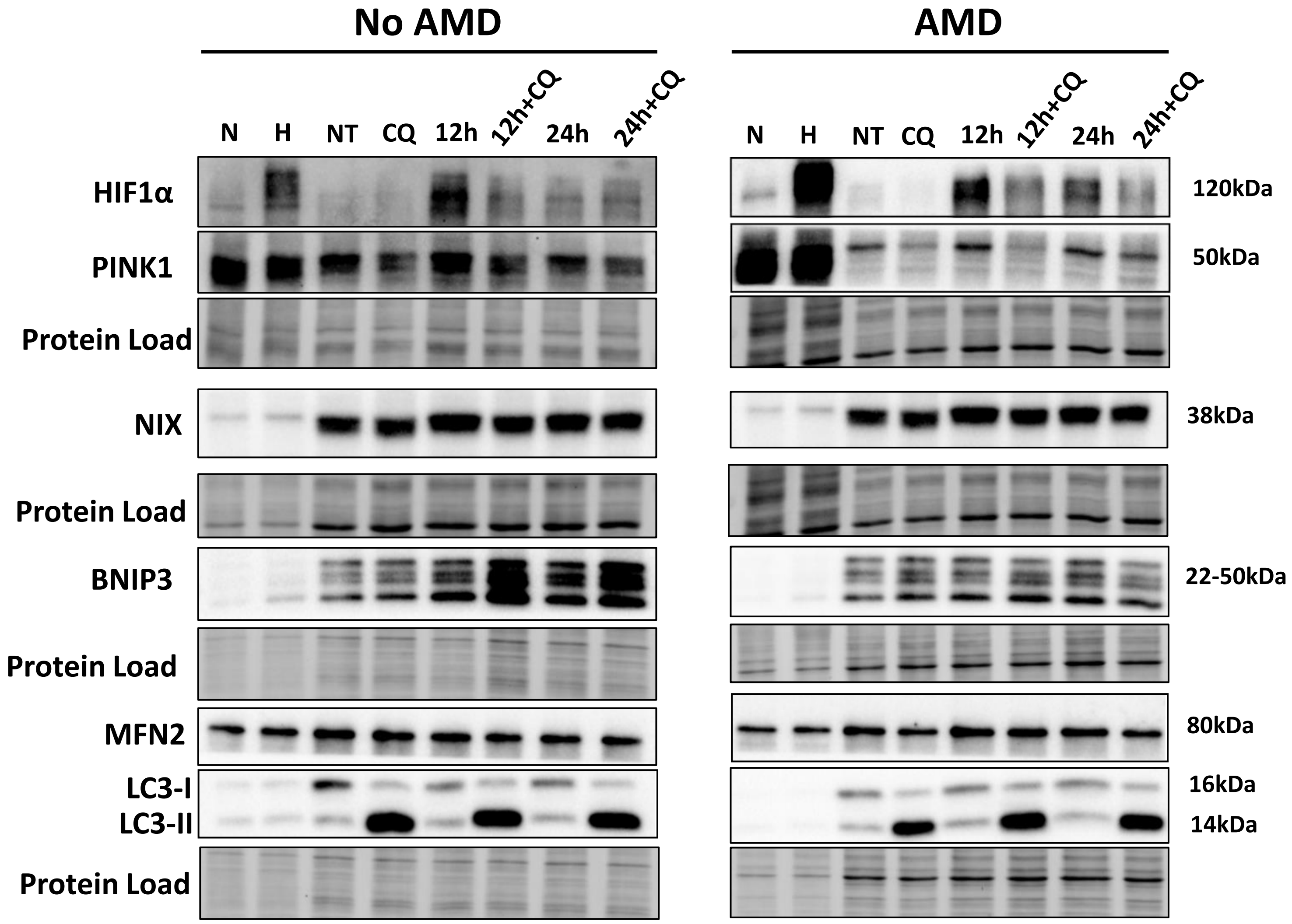


| |
|---|
| Normoxic |
| Hypoxic |
| NT |
| CQ |
| 12 CoCl2 |
| 12 CoCl2 + CQ |
| 24 CoCl2 |
| 24 CoCl2 + CQ |

Supplementary Figure 4.

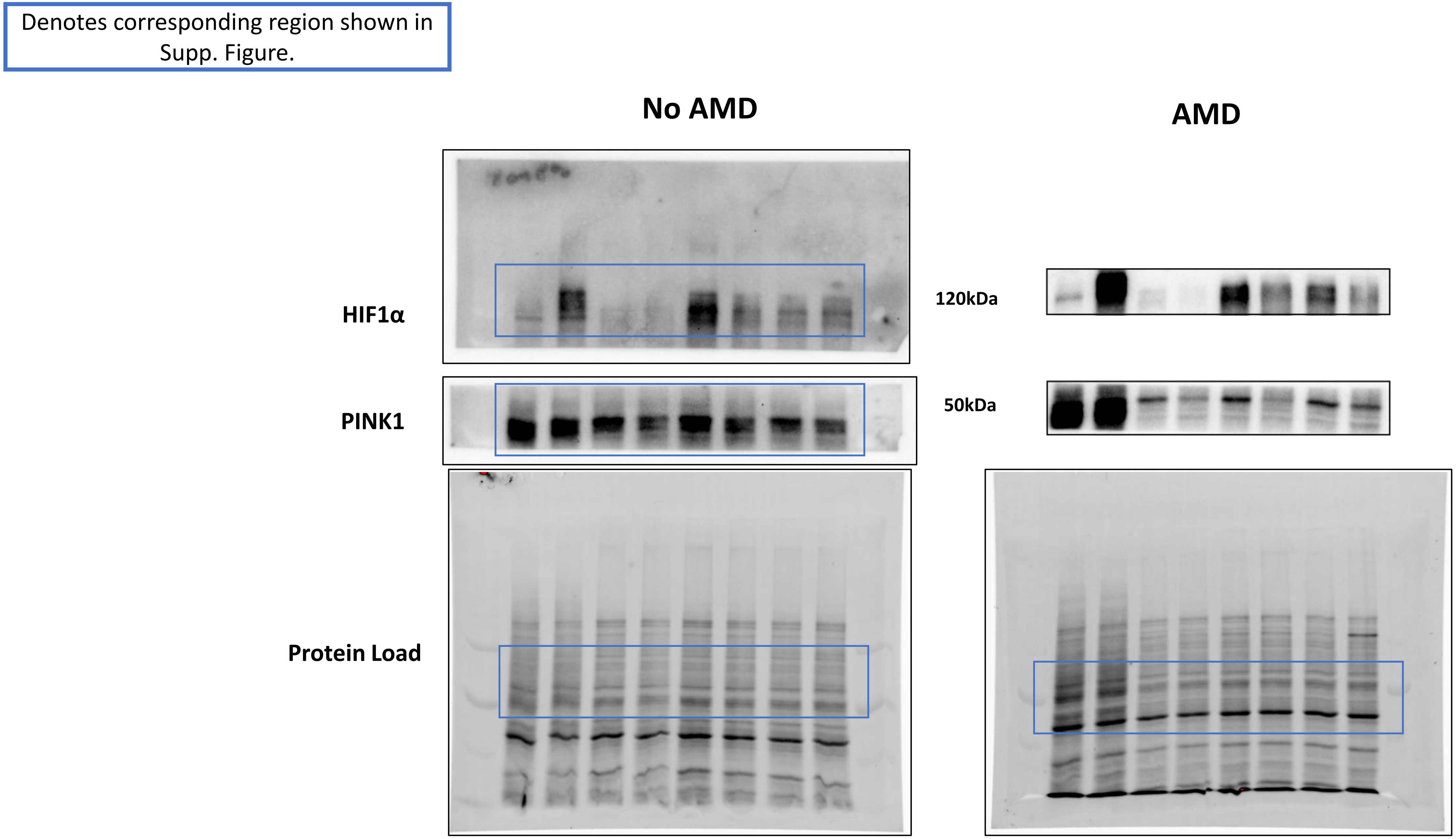

Supplementary Figure 4.

Denotes corresponding region shown in Supp. Figure.

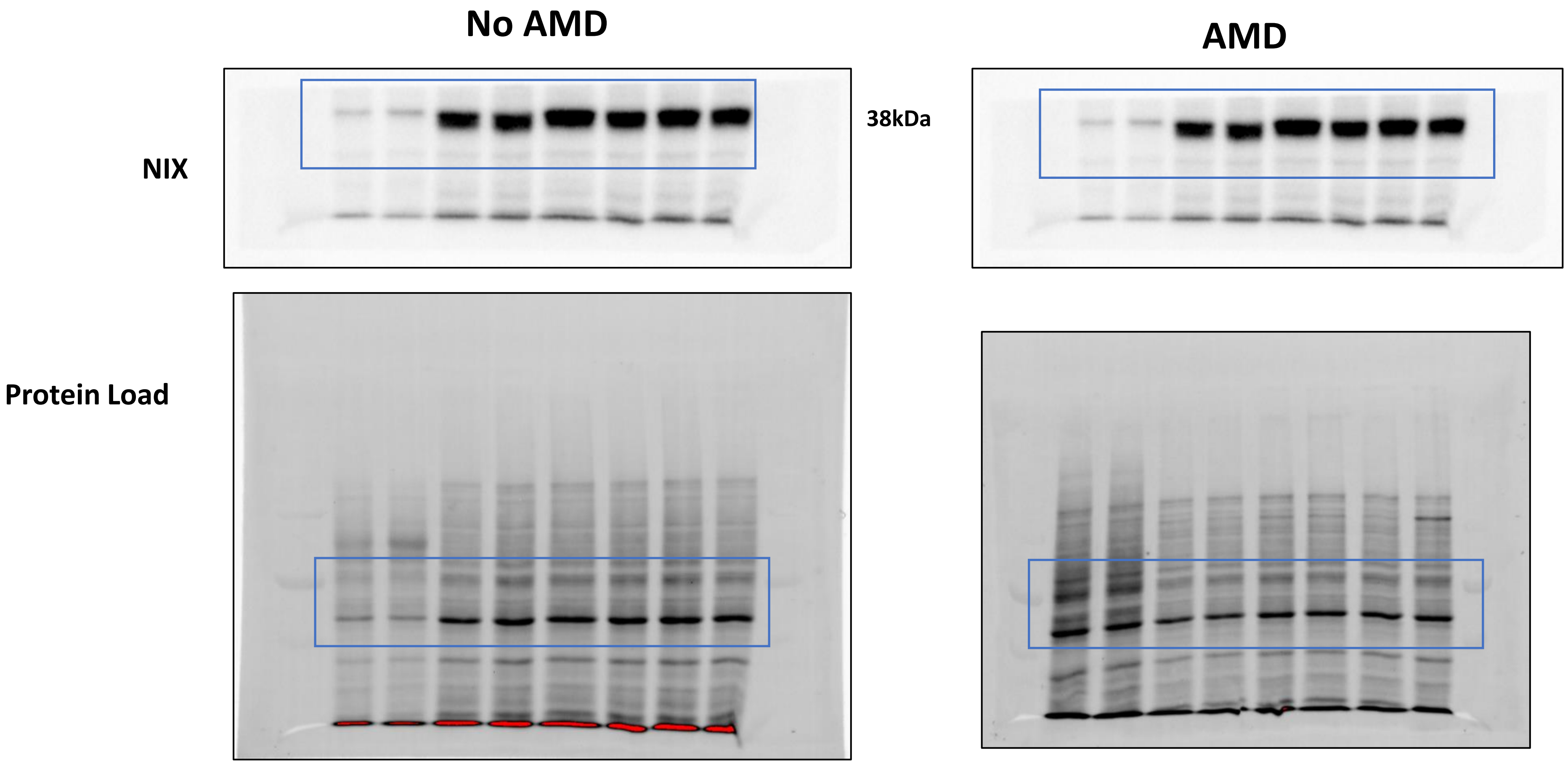

Supplementary Figure 4.

Denotes corresponding region shown in Supp. Figure.

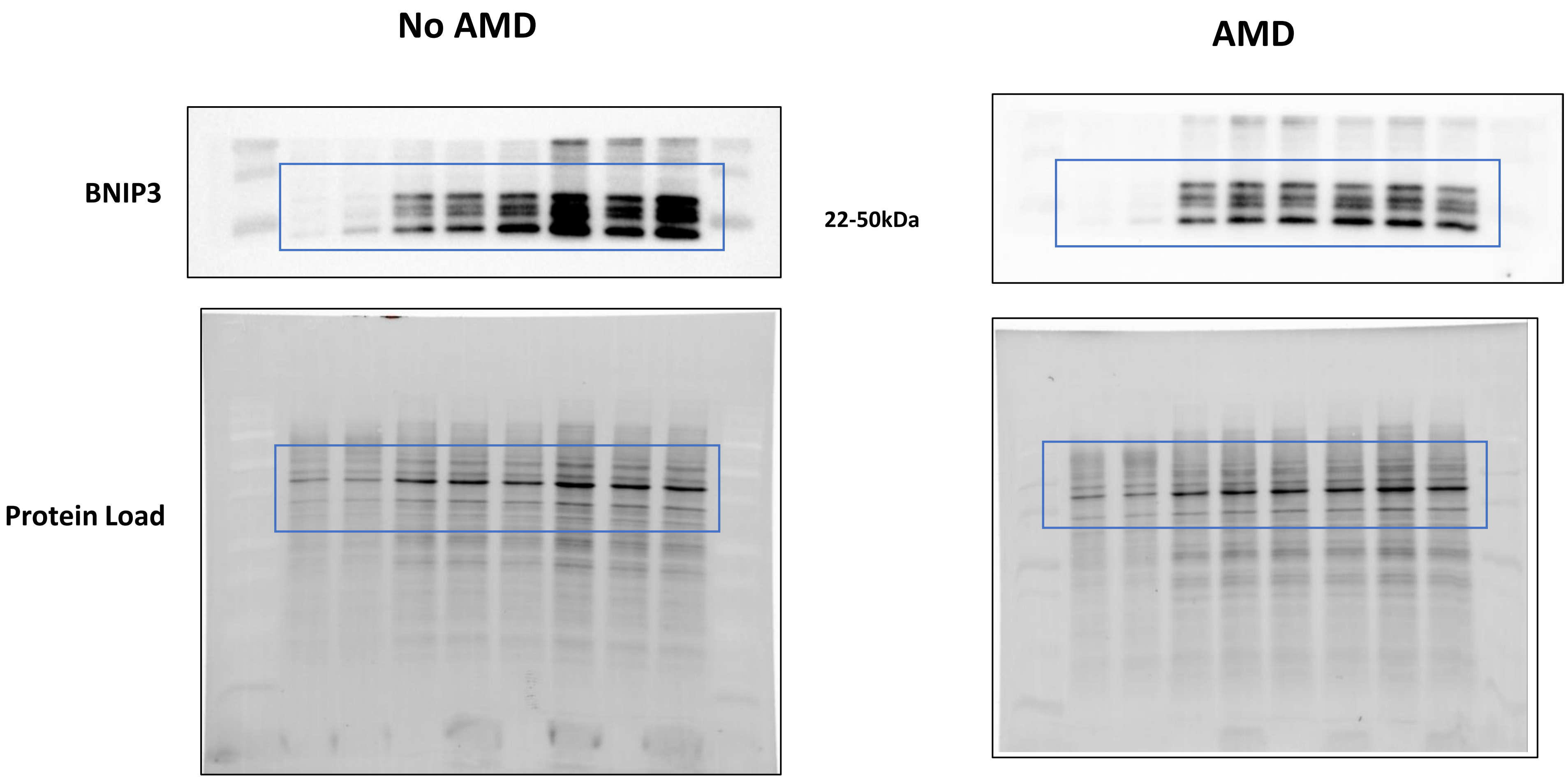

Supplementary Figure 4.

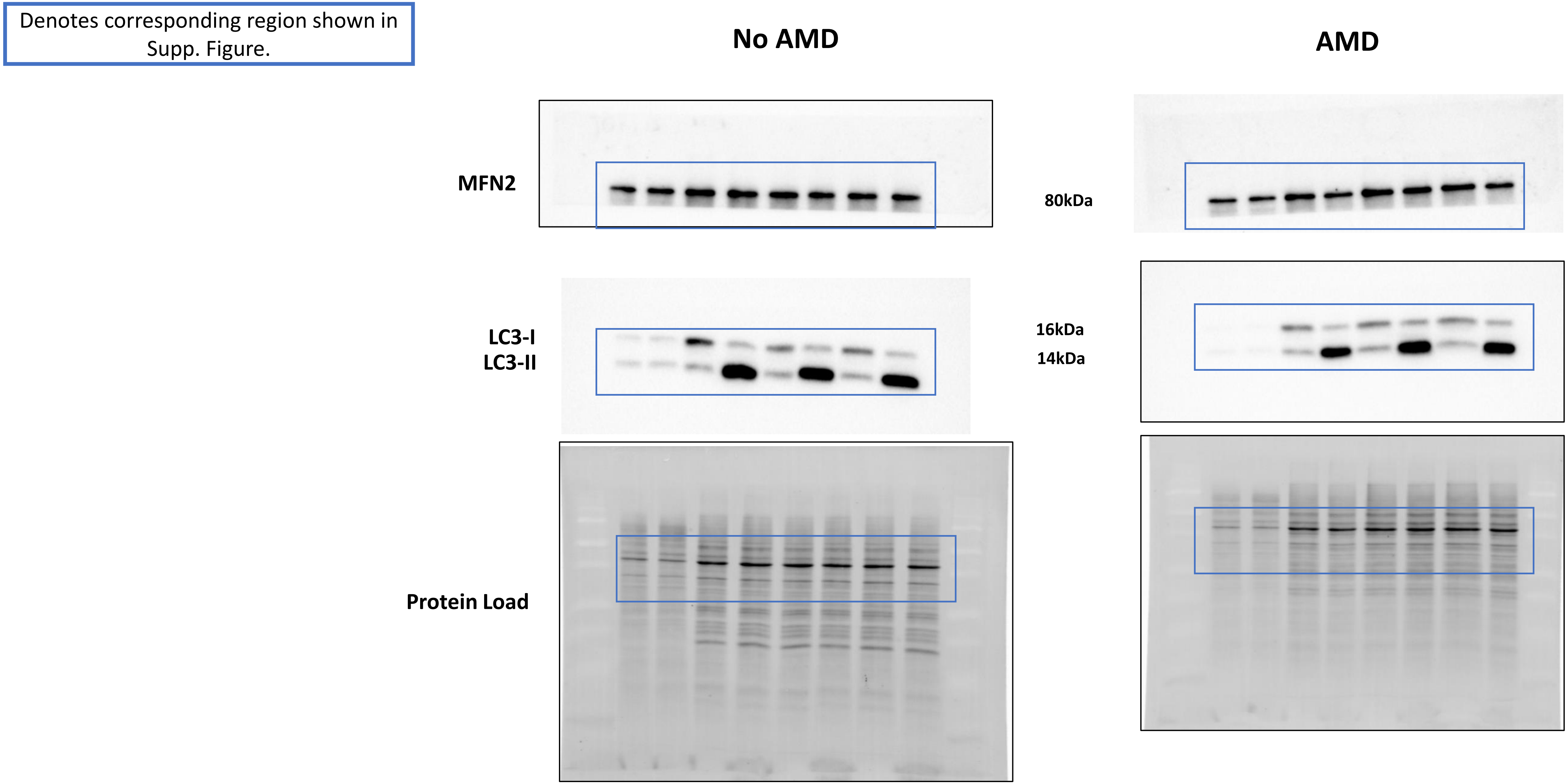

Supplement: Supplementary file 2 — Supplementary Information 2. [file 41598_2022_26012_MOESM2_ESM.pdf]
